# Role of tumor-associated neutrophils in regulation of tumor growth in lung cancer development: A mathematical model

**DOI:** 10.1371/journal.pone.0211041

**Published:** 2019-01-28

**Authors:** Yangjin Kim, Donggu Lee, Junho Lee, Seongwon Lee, Sean Lawler

**Affiliations:** 1 Department of Mathematics, Konkuk University, Seoul, Republic of Korea; 2 Mathematical Biosciences Institute, Ohio State University, Columbus, Ohio, United States of America; 3 Division of Mathematical Models, National Institute for Mathematical Sciences, Daejeon, Republic of Korea; 4 Department of neurosurgery, Harvard Medical School & Brigham and Women’s Hospital, Boston, Massachusetts, United States of America; Dartmouth College Geisel School of Medicine, UNITED STATES

## Abstract

Neutrophils display rapid and potent innate immune responses in various diseases. Tumor-associated neutrophils (TANs) however either induce or overcome immunosuppressive functions of the tumor microenvironment through complex tumor-stroma crosstalk. We developed a mathematical model to address the question of how phenotypic alterations between tumor suppressive N1 TANS, and tumor promoting N2 TANs affect nonlinear tumor growth in a complex tumor microenvironment. The model provides a visual display of the complex behavior of populations of TANs and tumors in response to various TGF-*β* and IFN-*β* stimuli. In addition, the effect of anti-tumor drug administration is incorporated in the model in an effort to achieve optimal anti-tumor efficacy. The simulation results from the mathematical model were in good agreement with experimental data. We found that the N2-to-N1 ratio (N21R) index is positively correlated with aggressive tumor growth, suggesting that this may be a good prognostic factor. We also found that the antitumor efficacy increases when the relative ratio (Dap) of delayed apoptotic cell death of N1 and N2 TANs is either very small or relatively large, providing a basis for therapeutically targeting prometastatic N2 TANs.

## Introduction

Lung cancer is the leading cause of cancer mortality worldwide, with an approximate 1.6 million deaths each year [1]. The most common (∼85%) form of lung cancer in patients is non-small cell lung cancer (NSCLC), of which lung squamous cell carcinoma (LUSC) and lung adenocarcinoma (LUAD) are the most common subtypes [2]. Various groups of myeloid cells have been known to promote tumor development by direct inhibition of immune responses [3], as well as by secreting growth factors, angiogenic factors, or matrix-degrading enzymes [4, 5]. For example, tumor-associated macrophages (TAMs), also known as M2 macrophages [3], have been shown to promote tumor growth [6, 7]. There is growing evidence suggesting that neutrophils play a major role in tumor progression from establishment of tumor formation and throughout the progression to the malignant state [8–12]. For example, tumor associated neutrophils (TANs) have been associated with poor prognosis in many cancers including metastatic melanoma [13], bronchoalveolar carcinoma [14], and renal carcinoma [15]. Like TAMs, TANs infiltrate tumor tissue and can have two differential states in cancer progression [8, 9, 16]: (i) an antitumorigenic role (called N1) (ii) promoting tumor progression (called N2). How these two phenotypes are regulated is largely unknown but many experimental and clinical findings suggest the significant potential of therapeutic targeting of the prometastatic role of TANs [17].

**Fig 1:**
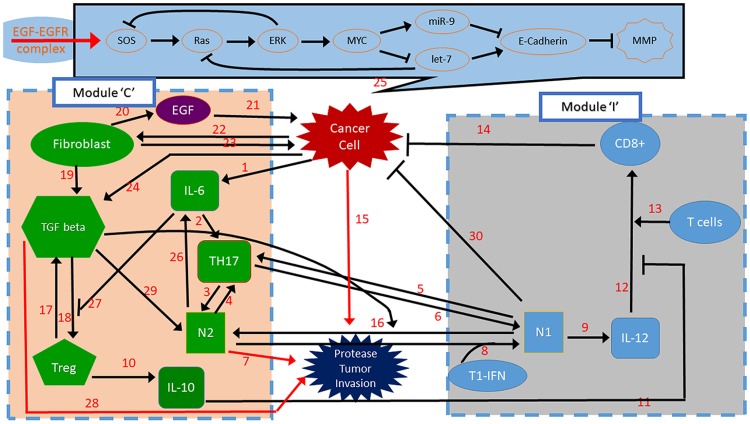
A schematic of tumor-microenvironment interaction. (Top) A signaling pathway for lung cancer. (Bottom) Network of interactions between cells involved (cancer cells, fibroblasts, Th17 cells, N1 cells, N2 cells, CD8+ T cells, Tregs) and cytokines and growth factors (EGF, IL-6, IL-10, IL-12, MMPs, TGF*β*). By convention, the kinetic interpretation of solid arrows and hammerheads in the interaction network represents induction (arrow) and inhibition (hammerhead), respectively. The module on the left and right will be represented by a module ‘C’ and ‘I’, respectively, in our simplified mathematical model. References for each interaction (number on the arrow or hammerhead) are provided in Table 1.

TGF-*β* has been identified as a major cytokine within a tumor that skews neutrophil differentiation toward the N2 phenotype [16, 18, 19], while TGF-*β* blockade and type-1 IFN (*α*, *β*, *ω*) treatment are known to shift the balance toward the N1 phenotype [20, 21]. IFN-*β* in tumor microenvironment can directly suppress tumor growth [22] by interacting with p53 [23–25]. IFN treated neutrophils were shown to upregulate PD-L1 and suppress T-cell proliferation [26]. After binding to interferon receptor type 1, IFNAR1 and IFNAR2, Type 1 IFN-*β* signals through TYK2 and JAK1, which in turn phosphorylate STAT family members (STAT1, STAT2, STAT3, and others) and activate its downstream functions to stimulate anti-tumor activities [27]. For example, vesicular stomatitis virus expressing IFN-*β* was shown to enhance anti-tumor immune responses in a murine model of NSCLC [28]. It is well established that cancer associated fibroblasts (CAFs) can promote tumor growth, aggressive invasion, and metastasis through mutual interaction in the tumor microenvironment [29, 30]. Fibroblast-secreted IFN-*β* was also able to restrict expression of the p53 RNA stabilizer, WIG1, and bring down mutant p53 RNA levels, thus suggesting an alternative therapeutic agent for mutant p53 positive lung cancer patients [31]. There are multiple levels of crosstalk between neutrophils and many cells including other immune cells and Th17 cells [32]. Neutrophils may express many important factors such as IL-6, IL-17A, IL-17F and IFN*γ* [33]. How neutrophils are induced by a tumor is still poorly understood. It is well known that tumor cells interact with stromal cells such as fibroblasts, immune cells (neutrophils, macrophages, Th17, Tregs, T cells), and cytokines in the tumor microenvironment, and that these complex interactions play a critical role in tumor initiation, growth, angiogenesis, and metastasis. The mutual interactions between a tumor and immune system involving TANs are summarized in Fig 1 with references in Table 1.

**Table 1:** Summary of interaction networks (Fig 1) in invasion processes in lung cancer development.

#	Description	References
1	cancer cells secrete IL-6	[157]
2	In combination with TGF-beta, IL-6 promotes the differentiation of Th17 cells by activating transcription factors including retinoic acid-related orphan receptor ROR*γ*t and ROR*α*	[158–160]
3	IL17 can indirectly induce the recruitment of neutrophils.	[32, 102]
4	Neutrophils secrete chemokines that can mediate the recruitment of Th17 cells and. secrete IL-17	[33, 102]
5, 6	IL17 can indirectly induce the recruitment of neutrophils	[32]
7	Neutrophils release TIMP-free MMP-9, MMP infiltration supplies MMP-9, Intravenous co-injection of mammary adenocarcinoma cells and neutrophils significantly raises lung metastases, N2 phenotype induces angiogenesis, invasion and metastasis, and maintain immunosuppression [161]	[161, 161–166]
8	Type I IFNs (or IFN*beta*) induce anti-tumor polarization, N2 TAN, in mice and human	[20, 21]
9	Neutrophils secrete/induce IL-12	[97, 99, 167]
10, 11	Tregs can inhibit tumor-specific CD8+ (54) and CD4+ (55) T cell effector functions through IL-10	[168–173]
12, 13	N1 Neutrophils promote recruitment and activation of CD8(+) cells by producing cytokines (IL-12, TNF*α*, GM-CSF, VEGF)	[97]
15, 23	Cancer associated fibroblasts (CAFs) promote tumor growth, aggressive invasion, and metastasis	[29, 30]
16	TGF-*β* within the tumor microenvironment induces TAN with a pro tumor phenotype. TGF-*β* blockade results in the recruitment and activation of TANs with an anti tumor phenotype	[16, 18, 19]
17	Tregs can inhibit tumor-specific CD8+ (54) and CD4+ (55) T cell effector functions through TGF*β*	[168–174]
18	TGF-*β* induces Foxp3+ T-reg from CD4+CD25	[160, 161, 175–177]
19	CAF secretes TGF-*β* and VEGF for Treg induction	[177, 178]
20, 21	CAF secretes EGF, which in turn promotes tumor growth and invasion	[41, 47, 64, 152, 178, 179]
22, 23	Lung cancer cells recruit CAFs and CAFs induce tumor growth, chemoresistance, angiogenesis, metastasis	[178]
24	Cancer cells change tumor microenvironment by secreting TGF-*β*	[178]
25	RAS-let-7-miR-9 signaling network induce lung cell invasion	[180–187]
26	N2 TANs expressed TGFbeta and IL-6	[8, 103, 104, 188]
27	IL-6 inhibits the generation of Treg cells and induces production of IL-17	[160]
28	IL-6 enhanced epithelial cell EMT and stimulated tumor progression by enhancing TGF*β* signaling. IL-6 and TGF*β* plays a central role in regulation of the paracrine loop between these two cytokines in LSCLS, TGF*β* exposure induces EMT in CSCs and non-CSCs	[189, 190]
29	“Neutrophils are driven by transforming growth factor-*β* (TGF*β*) to acquire a polarized, pro-tumoural N2 phenotype (characterized by high levels of arginase expression).”	[33]
30	Neutrophils have cytotoxic mediators for tumor cell killing such as TNF-*α*, IL-1*β*, and IFNs	[161, 191]

**Fig 2:**
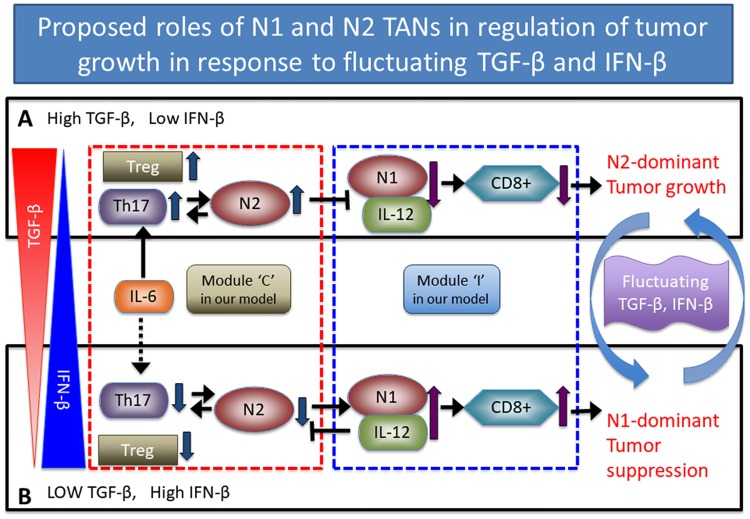
Proposed role of N1 and N2 complexes in the regulation of tumor growth and immune-suppression in response to fluctuating TGF-*β* and IFN-*β*. The phenotypic balance between N1 and N2 complexes determines tumor growth or suppression in response to TGF-*β* (red triangle on the left) and IFN-*β* (blue triangle on the left). (A) High TGF-*β* levels and low IFN-*β* activities induce Treg infiltration which in turn leads to biochemical suppression of the overall anti-tumor immune activities of T cells. TGF-*β* also modifies the tumor microenvironment by transforming N1 into N2 TANs [8, 34] while IFN-*β* brings the N2 phenotype back to the N1 phenotype [8]. This N2-dominant microenvironment leads to enhanced tumor growth. (B) Low TGF-*β* levels and upregulation of IFN-*β* reduce the phenotypical transition from N1 to N2 TANs and leads to upregulation of immune activities of T cells and N1 TANs, leading to suppression of tumor growth. Schematic components of the N2/Th17/Treg complex and the N1/IL12/CD8+ complex are represented by modules ‘C’ (box with the red dotted line) and ‘I’ (box with the blue dotted line) in our theoretical framework. Blue arrows on the right indicate the switching behavior between a tumor-promoting mode in (A) and the tumor-suppressive state in (B) in response to fluctuating TGF-*β* and IFN-*β* levels.

In this work, we employed the framework of the differential equation model in order to take into account the role of N1 and N2 TANs in the regulation of tumor-neutrophil interactions and tumor growth. Based on a schematic diagram in Fig 2, the model consists of a system of ordinary and delay differential equations involving the following variables:

$$\begin{array}{c} C(t)=\;\text{density}\;\text{of}\;\text{the}\;\text{N2}\;\text{complex}\;\text{at}\;\text{time}\;t\text{,}\\ I(t)=\;\text{density}\;\text{of}\;\text{the}\;\text{N1}\;\text{complex}\;\text{at}\;\text{time}\;t\text{,}\\ T(t)=\;\text{density}\;\text{of}\;\text{tumor}\;\text{cells}\;\text{at}\;\text{time}\;t\text{.} \end{array}$$

**Fig 3:**
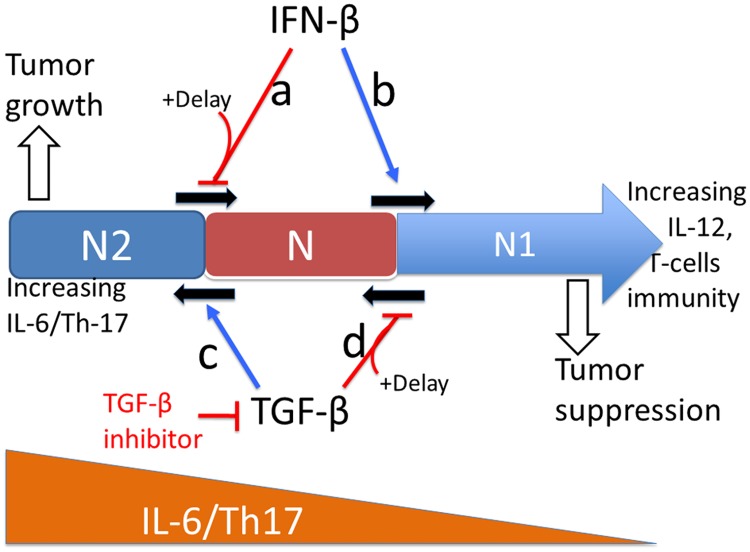
Tumor suppressive and tumor promoting roles of TGF-*β* and IFN-*β* respectively in the tumor microenvironment. As the CD8+ cytokines (IL-12) increase, differentiation of N cells transits to the N1 phenotype. There are three ways that IFN-*β*, TGF-*β* inhibitor, and delayed apoptotic pathways can keep the system in the safe-guard immune N1 zone. (Cases a, b) Increasing IFN-*β* levels induces the transition from N2 to N1-zone (case b) [22–25, 35–37]. The delayed apoptotic cell death of N2 TANs suppresses entry into the transition phase (case a). (Cases c, d) As the IL-6 level is increased (to the left), the tumor-promoting phenotype, N2, becomes dominant and TGF-*β* promotes the exit from the transition zone to enter the N2-zone (case c) [35]. The TGF-*β* inhibitor can reverse the phenotype back to the N1 microenvironment [38]. The delayed apoptotic cell death of N1 TANs suppresses entry into the transition phase (case d).

TGF-*β* and IFN-*β* play a central role in the critical transition between N1 and N2 TANs, resulting in either tumor-promoting or tumor-suppressing microenvironment. We summarize in Fig 3 the fundamental, phenotypic spectrum underlying how the oncogenic and tumor suppressive properties of TANs arise in relation to TGF-*β*, IFN-*β*, and delayed apoptotic cell death mechanisms. This model is then used (i) to investigate how TGF-*β* and IFN-*β* regulate the balance between N1 and N2 phenotypes by complex mutual interactions, (ii) to investigate how up- or down-regulation of these N1 and N2 phenotypes affect tumor growth and anti-tumor efficacy in the absence and presence of delayed apoptotic cell death mechanisms, and (iii) to develop optimal strategies of the combination therapy with TGF-*β* inhibitor and IFN-*β*.

## Materials and methods

### Mathematical model

#### N1 module and N2 complex

In this work, we assume: (i) High levels of TGF-*β* and IL-6 induce upregulation of the N2 complex [8, 34]. (ii) High IFN-*β* levels induce upregulation of the N1 phenotype [8]. (iii) Cells in the N1 and N2 complex have natural decay at rates *μ*_*N*1_, *μ*_*N*2_, respectively. (iv) Those factors in (i) and (ii) determines the critical transition between N1 and N2 complexes [35], determining either promotion or suppression of tumor growth.

**Fig 4:**
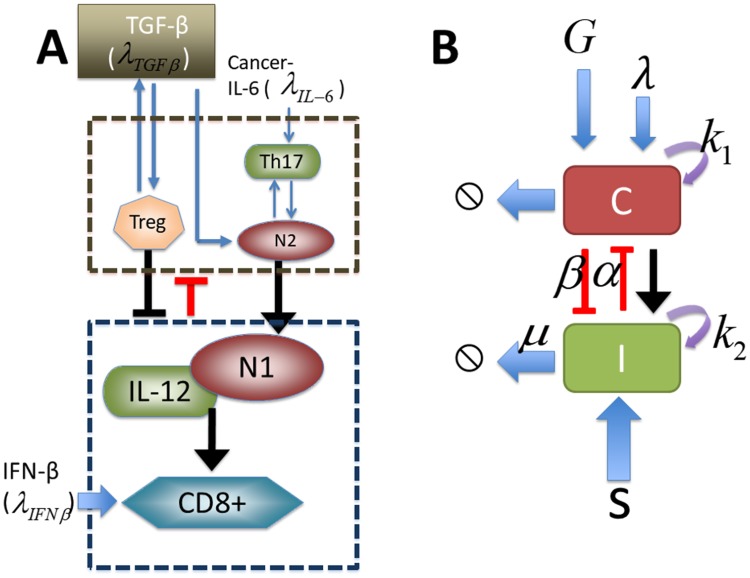
A schematic of our mathematical model.

In order to incorporate the signaling network into a mathematical model we simplified the network shown in Fig 4A as follows: We merged the N2 regulatory network among Treg, Th17 and N2 cells into one component (N2 complex; brown dashed box in Fig 4A) while we merged the immune regulatory network of N1 cells, IL-12 and CD8+ (N1 complex; blue dashed box in Fig 4A) in a separate module. The mathematical model network is shown in Fig 4B. By convention, the kinetic interpretation of solid arrows and hammerheads in the signaling network represents induction (arrow) and inhibition (hammerhead), respectively.

Let the variables *N*_2_(*t*) and *N*_1_(*t*) be activities of the N2 complex and N1 module, respectively, at time *t*. The scheme includes autocatalytic activities of the N2 complex (*N*_2_) and N1 complex (*N*_1_), removal of those key cells in the tumor microenvironment, mutual inhibition between those two modules, and clearance/decay of those complexes.

The mass balance (or conservation of mass) of a physical variable states that the rate of change of this quantity is the sum of contributing factors such as an input factor with the positive sign and an output factor with the negative sign. Therefore, the mass balance of the densities of the N2 module (*N*_2_) and N1 module (*N*_1_) leads to a system of ordinary differential equations (ODEs) as follows:

$$\frac{dN_{2}}{d\tau }=f_{1}\left(\lambda_{IL-6},g\right)+\frac{a_{1}a_{3}^{2}}{a_{3}^{2}+a_{4}F\left(N_{1}\right)}-\mu_{N2}N_{2},$$ (1)

$$\frac{dN_{1}}{d\tau }=f_{2}\left(s\right)+\frac{a_{2}a_{5}^{2}}{a_{5}^{2}+a_{6}H\left(N_{2}\right)}-\mu_{N1}N_{1}.$$ (2)

The first term in Eq (1) represents the signaling from N2-promoting molecules such as IL-6 and TGF-*β*. Similarly, the first term in Eq (2) represents the signaling from N1-promoting molecules such as IFN-*β*. The third terms in Eqs (1) and (2) indicate natural decay/consumption of N2 and N1 complexes, respectively. The second term in Eq (1) represents autocatalytic enhancement of the N2 module (numerator) with inhibition from the N1 module (denominator). The second term in Eq (2) is defined in a similar fashion. In more detail, *f*_1_(λ_*IL*−6_, *g*) is a positive function that represents the signaling pathways from cancer-induced IL-6 (λ_*IL*−6_) and cancer-induced TGF-*β* (*g*) to the N2 module, *f*_2_(*s*) is a function that represents the signaling pathways from IFN-*β* (*s*) to the N1 complex, *a*_1_, *a*_2_ are the autocatalytic enhancement parameters for the N2 and N1 modules, respectively, *a*_3_, *a*_5_ are the Hill-type inhibition saturation parameters from the counter part of N2 and N1 complex, respectively, *a*_4_ is the inhibition strength of the N2 complex by the N1 TANs, *a*_6_ is the inhibition strength of the N1 module by the N2 module, and finally *μ*_*N*2_, *μ*_*N*1_ are the clearance/death rate of N2 and N1 complexes, respectively.

As indicated in Eq (1), the signals λ_*IL*−6_ and *g* increase the rate of N2 activation through the function *f*(λ_*IL*−6_, *g*) in the first term, while N1-dependent inhibition of the N2 complex is expressed through the function *F*(*N*_1_) in the denominator of the second term. A simple requirement of these functions is that the function has to be an increasing function of the factor molecules. For example, the signaling function of the N2 complex (*f*_1_(λ_*IL*−6_, *g*)) increases as the signaling of IL-6 (λ_*IL*−6_) and TGF-*β* (*g*) increases, and the inhibition strength of the N2 complex by the N1 complex, *F*(*N*_1_), increases as the N1 population (*N*_1_) increases. In other words, $\frac{\partial f_{1}}{\partial \lambda_{IL-6}}>0,\;\frac{\partial f_{1}}{\partial g}>0$ for all non-negative λ_*IL*−6_ and *g*, and $\frac{\partial F}{\partial N_{1}}>0$ for all non-negative *N*_1_. Similarly, the first term *f*_2_(*s*) on the right-hand side of Eq (2) represents an increase in N1 activity induced by the signal *s*, and N2-dependent suppression of N1 activity is explored through the function *H*(*N*_2_) in the denominator. The signaling function of the N1 complex (*f*_2_(*s*)) increases as the signaling of IFN-*β* (*s*) increases, and the inhibition strength of N1 complex by N2 complex, *H*(*N*_2_), increases as the N2 population (*N*_2_) increases. In other words, $\frac{\partial f_{2}}{\partial s}>0,\;\frac{\partial H}{\partial N_{2}}>0$ for all non-negative *s*, *N*_2_. Based on biological observations (Fig 4A), we assume that

$$\begin{array}{ll} f_{1}\left(\lambda_{IL-6},g\right)=\lambda_{IL-6}+\lambda_{TGF-\beta }g, & F\left(N_{1}\right)=N_{1}^{2},\\ f_{2}\left(s\right)=\lambda_{IFN\beta }s, & H\left(N_{2}\right)=N_{2}^{2}, \end{array}$$ (3)

where λ_*TGF*−*β*_ is the TGF-*β*-induced signal rate and λ_*IFNβ*_ is the IFN-*β* -induced signal rate.

After performing the following non-dimensionalization

$$\begin{array}{ccc} & & t=\mu_{N2}\tau ,\;C=\frac{N_{2}}{N_{2}^{*}},\;I=\frac{N_{1}}{N_{1}^{*}},\;\lambda =\frac{\lambda_{IL-6}}{\mu_{N2}N_{2}^{*}},\;G=\frac{g}{G^{*}},\;\lambda_{G}=\frac{\lambda_{TGF-\beta }G^{*}}{\mu_{N2}N_{2}^{*}},\\ & & \mu =\frac{\mu_{N1}}{\mu_{N2}},\;S=\frac{s}{S^{*}},\;\lambda_{S}=\frac{\lambda_{IFN\beta }S^{*}}{\mu_{N2}N_{1}^{*}},\;k_{1}=\frac{a_{1}a_{3}^{2}}{\mu_{N2}N_{2}^{*}},\;k_{3}=a_{3},\;k_{2}=\frac{a_{2}a_{5}^{2}}{\mu_{N2}N_{1}^{*}},\\ & & \;k_{4}=a_{5},\;\alpha =a_{4}{\left(N_{1}^{*}\right)}^{2},\;\beta =a_{6}{\left(N_{2}^{*}\right)}^{2}, \end{array}$$ (4)

from the dimensional Eqs (1)–(3), we obtain the associated dimensionless equations for N2 (*C*) and N1 (*I*) complexes with a set of essential control parameters:

$$\frac{dC}{dt}=\lambda +\lambda_{G}G+\frac{k_{1}}{k_{3}^{2}+\alpha I^{2}}-C,$$ (5)

$$\frac{dI}{dt}=\lambda_{S}S+\frac{k_{2}}{k_{4}^{2}+\beta C^{2}}-\mu I.$$ (6)

#### Tumor volume (*T*(*t*))

As we discussed above, neutrophils play either tumorigenic or anti-tumorigenic roles in response to various biochemical signals such as TGF-*β* and IFN-*β* in a complex microenvironment. While N1 TANs kill tumor cells [8], N2 TANs promote tumor growth [8, 35]. Therefore, tumor growth and tumor cell killing are promoted or suppressed by relative balance between N1 and N2 TANs [8], which, in our model, is determined by dichotomous polarization of neutrophils (either N2 TANs (*C*) or N1 TANs (*I*)) in Eqs (3)–(6) in response to microenvironmental signals such as IL-6 (λ), TGF-*β* (*G*) and IFN-*β* (*S*). Gompetz growth, logistic growth, and other nonlinear growth curves have been suggested to fit to the experimental data on tumor growth [39]. Logistic growth in the absence and presence of growth factor-mediated enhancement of tumor growth has been observed in experiments [40–44] and mathematical models were successfully applied to predict these experimental results [7, 40–51]. We assume that (i) the tumor grows at a basic rate *r* in the absence of promotion of N2 TANs (*C*) and tumor cell killing by N1 TANs (*I*), following logistic growth, (ii) tumor growth is enhanced in a N2-mediated tumor microenvironment, (iii) anti-tumorigenic N1 TANs kill the tumor cell at a rate *δ*. In particular, the N2-mediated enhancement of tumor growth is modeled by a fraction of the N2 phenotype (*C*) relative to the N1 phenotype (*I*) in tumor microenvironment:

$$\frac{C}{K+\gamma_{1}I}$$

where *K*, *γ*_1_ are the inhibition parameters of N2-mediated growth.

The mass balance of the tumor volume (*T*) leads to an ordinary differential equation as follows:

$$\frac{dT}{dt}=r(1+\frac{C}{K+\gamma_{1}I})T(1-\frac{T}{T_{0}})-\delta \;I\;T,$$ (7)

where *r* is a growth rate of the tumor and *T*_0_ is the carrying capacity of the tumor population.

All simulations were performed using Matlab (Mathworks) for ordinary differential equations (ode45) and delay differential equations (dde23).

### Parameter estimation

*μ*_*N*1_, *μ*_*N*2_: Neutrophil turnover in blood is quite rapid [52]. The half-life of neutrophils within the circulation (*T*_1/2_) was reported to be 3.2 hours in rabbits [53, 54], 11.4 hours in mice [55], and 7-10 hours in humans [56, 57]. On the other hand, the measured half-life in *in vivo* experiments [58] was measured to be longer: 90 *h* in vivo experiments [59], 20.3 in neutropenia patients [60], and 17.3 *h* in terminal cancer patients with glioblastoma and chronic lymphocytic leukemia [61]. For example, Andzinski *et al*. [62] recently reported that the life span of TANs can be remarkably prolonged in tumor microenvironment where IFN-*β* activity is suppressed [63]. Based on these biological observations in the tumor microenvironment, neutrophils in tumor tissue, we take the half-life of about *T*_1/2_ = 20 *h*. In general, we can obtain the decay rate of a variable when we know the half-life. In more detail, by solving a typical differential equation of the form $\frac{dy}{dt}=-\lambda y$ with an initial condition *y*(0) = *y*_0_ and a delay rate λ, and comparing the solution *y*(*t*) = *y*_0_*e*^−λ*t*^ at *t* = *T*_1/2_, we can obtain the decay rate $\lambda =\frac{\ln(2)}{T_{1/2}}$. This calculation then leads to $\mu_{N2}=\frac{ln(2)}{20h}∼0.035h^{-1}$. We also take *μ*_*N*1_ = *μ*_*N*2_.

*μ*_*G*_: The decay rate of TGF-*β* was estimated to be 8.02 × 10^−6^ *s*^−1^ = 2.89 × 10^−2^ *h*^−1^ = 6.929 × 10^−1^ *day*^−1^ [7, 41, 47, 64]. Thus, dimensionless *μ*_*G*_ = 2.89 × 10^−2^ *h*^−1^/(0.035*h*^−1^) = 0.826.

*μ*_*L*_ (decay rate of TGF-*β* inhibitor): The half-life of galunisertib, TGF-*β* inhibitor, was measured to be 0.53 *h* in rat, 0.3 *h* in mice, 2.26*h* in dogs [65]. We take *μ*_*L*_ = 3 *h*, leading to *μ*_*L*_ = *ln*(2)/(3*h*) = 2.31 × 10^−1^ *h*^−1^. Thus, dimensionless *μ*_*L*_ = 2.31 × 10^−1^ *h*^−1^/(0.035*h*^−1^) = 6.6.

*μ*_*S*_ (decay rate of IFN-*β*): The serum half-life of IFN-*β* ranges from minutes to hours depending on biological conditions of its receptors in response to intravenous injection in mice [66]. The distribution and terminal half-lives were measured to be 5 *min* and 5 *h* in healthy male volunteers (N = 12) [67]. We take *μ*_*S*_ = 5 *h*, leading to *μ*_*S*_ = *ln*(2)/(5*h*) = 1.386 × 10^−1^ *h*^−1^. Thus, dimensionless *μ*_*S*_ = 1.386 × 10^−1^ *h*^−1^/(0.035*h*^−1^) = 3.96.

G*: The TGF-*β* level in the lung cancer microenvironment was measured to be in the range of 400 − 2, 000 *pg*/*mL* [38]. We take G* = 1, 000 *pg*/*mL*.

S*: Deng *et al*. [68] investigated the effect of STING-dependent cytosolic DNA sensing on the IFN-*β*-dependent anti-tumor efficacy with the control IFN-*β* concentration of 10 *ng*/*mL* and IFN*γ* value of 20 *ng*/*mL*. We take S* = 10 *ng*/*mL*.

Table 2 summarizes all the essential parameter values in the system (5)–(7).

**Table 2:** Parameters used in the model.

Par	Description	Value	Refs
N1/N2 modules			
λ	IL-6-induced signaling source of the N2 module	0.01	[103], Estimated
*G*	TGF-*β*-induced signaling source of the N2 module	0-1.0	[38]
*k*_1_	autocatalytic production rate (N2 module)	4.0	Estimated
*k*_3_	Hill-type coefficient	1.0	Estimated
*α*	Inhibition strength of the N2 module by the N1 module	1.5	Estimated
*k*_2_	autocatalytic production rate (N1 module)	4.0	Estimated
*k*_4_	Hill-type coefficient	1.0	Estimated
*β*	Inhibition strength of the N1 module by the N2 module	1.0	Estimated
*S*	IFN-*β*-induced signaling source of the N1 module	0-1.0	[68], Estimated
*μ*	Relative decay rate of N1 and N2 TANs	1.0	[60, 61]
*th*_*C*_	Threshold of the N2 module for tumor growth switch	1.5	Estimated
Tumor module			
*r*	Tumor growth rate	0.05	[36]
*K*	Inhibition parameter of N2-mediated growth	1.0	[36], Estimated
*γ*_1_	Inhibition parameter of N2-mediated growth	0.1	[36], Estimated
*T*_0_	Carrying capacity of a tumor	100	[36], Estimated
*δ*	Killing rate of tumor cells by N1 TANs	0.005	[36], Estimated
Therapeutics			
*G*_*s*_	TGF-*β* source	0.826	Estimated
*μ*_*G*_	Decay rate of TGF-*β*	0.826	[7, 41, 47, 64].
*γ*_*L*_	Clearance rate of TGF-*β* from TGF-*β* inhibitor binding	100	Estimated
*L*_*s*_	Injection rate of TGF-*β* inhibitor	13	Estimated
*μ*_*L*_	Decay rate of TGF-*β* inhibitor	6.6	[65]
*S*_*S*_	IFN-*β* injection rate	1 -100	[22–25, 36, 37]
*μ*_*S*_	Decay rate of IFN-*β*	3.96	[67]
Reference values			
G*	TGF-*β* Concentration	1 *ng*/*mL*	[38]
S*	IFN-*β* Concentration	10 *ng*/*mL*	[68]
T*	Tumor Volume	100 *mm*^3^	[36]

## Results

### Dynamics of the model

**Fig 5:**
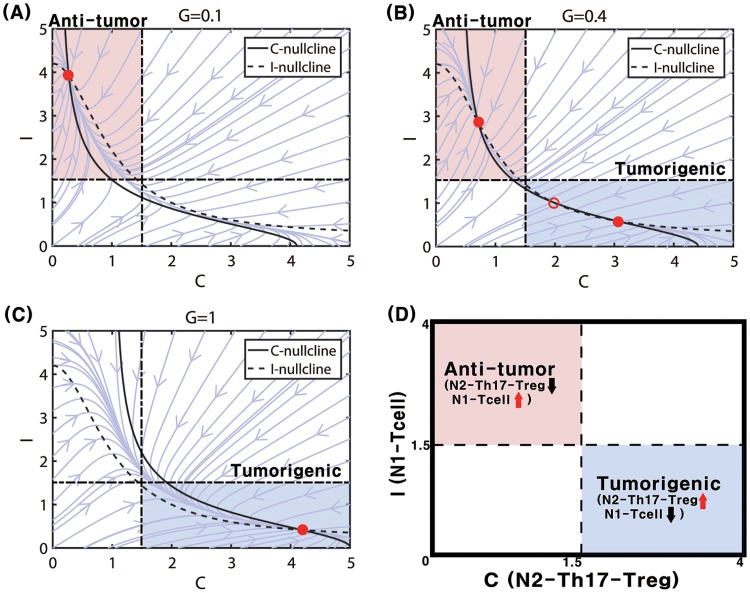
Nonlinear dynamics. Dynamics of the system (5) and (6) in the C-I phase plane in response to low (*G* = 0.1 in (A)), intermediate (*G* = 0.4 in (B)), and high (*G* = 1.0 in (C)) levels of TGF-*β*-induced transition signals. Steady states marked with circles (stable steady state = filled circle, unstable steady state = empty circle). (D) A schematic of anti-tumorigenic (*C* < *C*^*th*^, *I* > *I*^*th*^) and tumorigenic (*C* > *C*^*th*^, *I* < *I*^*th*^) regions in the *C* − *I* plane. *S* = 0.2 fixed. Other parameters are given in Table 2.

We first investigated the local dynamics of the system (5) and (6). By taking different initial values of N1 and N2 complexes and numerically solving Eqs (5) and (6), one can obtain solutions (*C*(*t*), *I*(*t*)) at time *t* and plot the relative locations of solutions in the *C* − *I* domain. In Fig 5 we illustrate three different flow patterns of the system (5) and (6) near the equilibrium point (steady state (SS); circles) in response to low (*G* = 0.1 in Fig 5A), intermediate (*G* = 0.4 in Fig 5B), and high (*G* = 1.0 in Fig 5C) TGF-*β* levels in the *C* − *I* phase diagram. We note that if the equilibrium point is *stable*, the solution converges to the stable SS (called *attractor*). On the other hand, when the equilibrium point is *unstable*, the solution does not converge to the unstable SS and the solution is repelled by the unstable SS (called *repeller*). By taking the thresholds, *C*^*th*^ (= 1.5) for the N2 level and *I*^*th*^ (=1.5) for the N1 level, we shall define the anti-tumorigenic ($P_{a}$) and tumorigenic ($P_{t}$) phases as follows:

$$\begin{array}{c} P_{a}=\left\{\left(C,I\right)\in R^{2}:C<C^{th};I>I^{th}\right\}\\ P_{t}=\left\{\left(C,I\right)\in R^{2}:C>C^{th};I<I^{th}\right\}. \end{array}$$

Fig 5D illustrates the anti-tumorigenic (low activities of the N2 module, high activities of N1 TANs) and tumorigenic (high activities of the N2 complex, low activities of the N1 complex) in a *C* − *I* plane. A low TGF-*β* level (*G* = 0.1) induces only one stable SS (Fig 5A) where suppression of N2 activities and promotion of N1 activities induce the anti-tumorigenic status ($P_{a}$). In this case, the environment eventually induces the anti-tumorigenic phenotype regardless of initial status of N1 and N2 complexes. In contrast, the increased activity of N2 TANs, and decreased activity of N1 TANs, leading to the tumorigenic phase ($P_{t}$), are induced under a high TGF-*β* condition (*G* = 1.0; Fig 5C; one stable SS). In this case, the environment eventually induces the tumorigenic phenotype regardless of initial status of N1 and N2 complexes. For an intermediate level of TGF-*β* signals (*G* = 0.4), there exist three equilibria: two stable SS (two filled circles; one in $P_{t}$ and one in $P_{a}$) and one unstable SS (empty circle) in the middle. This leads to a bi-stable system shown in Fig 5B. In this case, the environment will induce either anti-tumorigenic or tumorigenic phenotype depending on the initial status of N1 and N2 complexes. From these observations, we anticipate to see a bifurcation curve, especially hysteresis, *i.e.,* the N2 level with TGF-*β* signal parameter *G*.

### Effect of TGF-*β* on transitions between N1/N2 TANs and tumor growth

**Fig 6:**
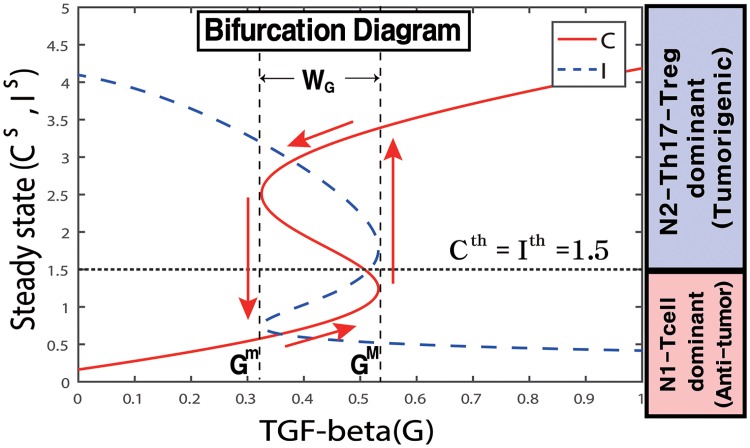
Bifurcation diagram. High and low TGF-*β* signals (*G*) provide an on-off switch of N2 activation and determine the dichotomous behavior: cancer cell activation and benign status. Y-axis = steady state (SS) of levels of the N2 (*C*) and N1 (*I*) complex modules. *W*_*G*_ = [*G*^*m*^, *G*^*M*^] = a window of bi-stabilty. *S* = 0.2. Other parameters are given in Table 2.

When the system (5) and (6) is in equilibrium, we can solve *C* as a function of the TGF-*β* signal (*G*). Fig 6 shows the graphs *C* = *C*(*G*) and *I* = *I*(*G*) as a hysteresis. While the upper and lower branches are stable, the middle branch is unstable (recall the *G*-dependent stability in Fig 5). If *G* is small, then the system is in the lower branch (*C* low, *I* high), leading to the anti-tumorigenic phase ($P_{a}$). This situation continues to hold as *G* is increased until it reaches the right knee point of the curve (when *G* = 0.53). At this point, the N2 level jumps to the high branch, with an elevated level of N2 TANs, leading to the tumorigenic phase ($P_{t}$). As *G* is decreased, the N2 level remains elevated, until *G* is decreased to the left knee point of the curve (when *G* = 0.33), at which time the N2 level jumps down to the lower branch, and the system returns to the cancer-free mode ($P_{a}$). We conclude that the effect of *G* is history dependent: when *G* is at an intermediate level (0.33 = *G*^*m*^ < *G* < *G*^*M*^ = 0.53; bi-stable mode in Fig 5B), the system induces the anti-tumor phase $P_{a}$ if *G* is in an increasing mode from the low *G* value, and leads to the tumor-promoting phase $P_{t}$ if *G* is in decreasing mode from the high *G* level. The bifurcation diagram in Fig 6 suggests that a state (*G*, *C*) with *C* > *C*^*th*^ = 1.5 will be moved by the dynamical system (5) and (6) into the upper stable steady state branch, resulting in prevailing $P_{t}$-status. On the other hand, if *C* < *C*^*th*^ = 1.5, then (*G*, *C*) will stay in the lower stable state branch, leading to $P_{a}$-status. The size of the bi-stability window (*W*_*G*_ = [*G*^*m*^, *G*^*M*^]) depends on other parameters and may even disappear for some parameter sets.

**Fig 7:**
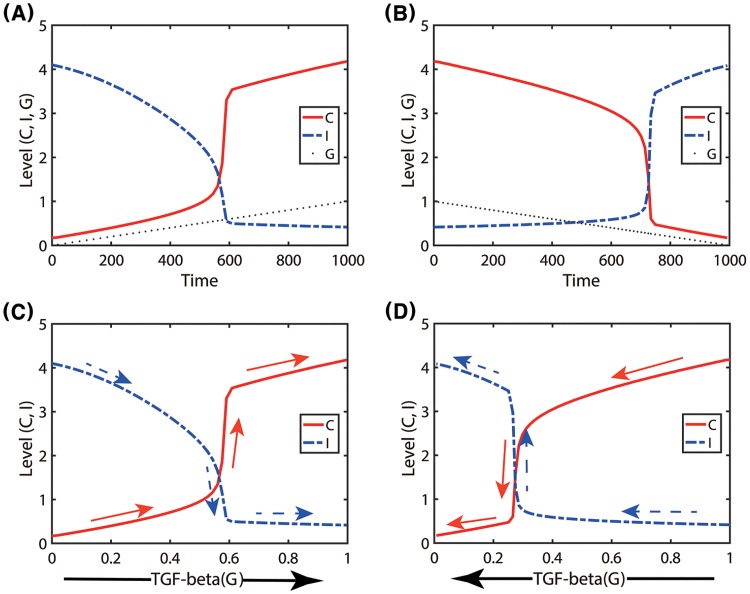
Immune response of TANs to increasing or decreasing TGF-*β*. (A, B) Time courses of the N2 (*C*, red solid curve) and N1 (*I*, blue dashed curve) levels as the TGF-*β* level (*G*; black dotted line) is slowly increased (from 0 to 1.0 in (A)) and slowly decreased (from 1.0 to 0.1 in (B)), respectively. (C, D) Change of *C* and *I* as a function of *G* corresponding to (A, B), respectively. Arrows indicate the moving direction of the solutions. *S* = 0.2. Other parameters are given in Table 2.

Fig 7A shows how main variables (*C*, *I*) in the system respond to the slowly increasing TGF-*β* level (*G*). The solution *C*(*t*) (solid red line) initially follows the $P_{a}$-status along the lower branch of the bifurcation curve until the right knee point of the bifurcation curve and transits to the upper branch solution, leading to the $P_{t}$-mode. This trajectory of the solutions (*C*, *I*) along *G* is illustrated in Fig 7C. This illustrates that when *G* is increasing from a low value, the system will stay longer in the $P_{a}$-phase. On the other hand, when *G* is slowly decreased from a high value, the solution *C*(*t*) follows the upper branch of the bifurcation curve in Fig 6 until the left knee point and drops down to the lower branch, staying longer in the $P_{t}$-phase including the bistability region *W*_*G*_ (Fig 6). The corresponding trajectory of the solutions (*C*, *I*) along *G* is shown in Fig 7D.

**Fig 8:**
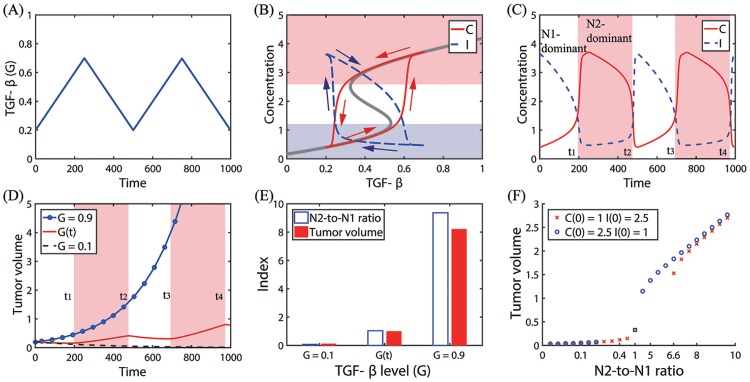
Effect of fluctuating TGF-*β* on tumor growth. (A) A time-dependent TGF-*β* level was assigned for a periodic supply of TGF-*β* in tumor microenvironment. (B) Trajectories of solutions ((*G*(*t*), *C*(*t*)) and (*G*(*t*), *I*(*t*))) in response to TGF-*β* in (A). The thick gray curve represents the upper and lower branches of steady states (*C* − *G* hysteresis bifurcation loop in Fig 6). Red and blue arrows = solution flow direction of *C* and *I*, respectively. (C) Time courses of concentrations of *C* and *I* in response to periodic *G* input in (A). The N2-dominant $P_{t}$-regions were shaded in pink. (D) Time courses of tumor volumes in response to a fixed (*G* = 0.9 (blue circle); *G* = 0.1 (dashed)) and fluctuating TGF-*β* level. Fluctuating TGF-*β* levels induce transitions between N1- and N2-dominant phenotypes, leading to transient tumor growth at the transition times (*t*_1_, *t*_2_, *t*_3_, *t*_4_). (E) The N2-to-N1 ratio and tumor size for three TGF-*β* conditions in (D). (F) Positive correlations between the tumor volume and the N2-to-N1 ratio with two initial conditions (*C*(0) = 1, *I*(0) = 2.5 in red asterisk; *C*(0) = 2.5, *I*(0) = 1 in blue circle). Initial conditions in (A-E): *C*(0) = 0.4, *I*(0) = 3.6, *G*(0) = 0.2, *T*(0) = 0.2. Other parameters are given in Table 2.

In Fig 8, we test the effect of fluctuating TGF-*β* on tumor growth. A time-dependent TGF-*β* level was assigned for a periodic supply of TGF-*β* in tumor microenvironment as follows (Fig 8A):

$$\begin{array}{c} G\left(t\right)=\{\begin{array}{cc} 0.002t+0.2 & \text{if}\;0<t<250\\ -0.002(t-250)+0.7 & \text{if}\;250\leq t<500\\ 0.002(t-500)+0.2 & \text{if}\;500\leq t<750\\ -0.002(t-750)+0.7 & \text{if}\;750\leq t<1000. \end{array} \end{array}$$

In response to the initial increasing TGF-*β* level, the trajectory of the solution *C*(*t*) follows the lower branches of the hysteresis bifurcation loop (thick gray curve) and jumps to the upper branch (Fig 8B). However, a decrease in *G* around *t* = 250 switches the moving direction of the solution and it follows the upper branch in the bi-stability region until it jumps down to the lower branch near the left knee of the hysteresis curve. This flow of the solution (*G*(*t*), *C*(*t*)) was marked in red arrows with initial position on the left lower corner (Fig 8B). The blue dashed curve and blue arrows represent the solution (*G*(*t*), *I*(*t*)) and its flow, respectively, in the opposite side (Fig 8B). The detailed time courses of solutions (*C*(*t*), *I*(*t*)) in response to the periodic *G* input are shown in Fig 8C. The specific N1/N2 phenotypic transitions occur at time *t* = *t*_1_, *t*_2_, *t*_3_, *t*_4_. For example, the initial N1-dominant $P_{a}$-status (white region in Fig 8C) switches to a N2-dominant $P_{t}$-mode (pink region in Fig 8C) in the tumor microenvironment at time *t* = *t*_1_ due to increasing TGF-*β* levels. The TAN phenotypic ratio, described by $\frac{C}{K+\gamma_{1}I}$ in the second term of Eq (7), and N1 TAN immunity on tumor cells, described by *δIT* in the third term of Eq (7), determine either promotion or suppression of tumor growth in the model. Fig 8D shows time courses of tumor volumes in response to either fixed or varying TGF-*β*: (i) high TGF-*β* (*G* = 0.9, blue circle), (ii) low TGF-*β* (*G* = 0.1, dashed), and (iii) fluctuating TGF-*β* levels given in Fig 8A. The high TGF-*β* level induces N2-dominant $P_{t}$-phase (Fig 5C), therefore, leading to N2-mediated, faster tumor growth (blue circle). When *G* is low, the system induces the N1-dominant $P_{a}$ microenvironment (Fig 5A), resulting in N1-mediated, slower tumor growth (black dashed). On the other hand, fluctuating TGF-*β* induces alternating transitions between $P_{a}$ (white region) and $P_{t}$ (pink region) phases at *t* = *t*_1_, *t*_2_, *t*_3_, *t*_4_ (Fig 8C), which induces slow (white band in Fig 8D) or fast (pink band in Fig 8D) tumor growth in $P_{a}$ and $P_{t}$-regions, respectively, and eventually leads to an intermediate tumor volume at final time (Fig 8D). These results are in good agreement with experimental observations where N1 TANs significantly reduced the size of a tumor relative to N2 TANs [8]. The N2-to-N1 ratio (N21R) index and tumor volume corresponding to these three cases are shown in Fig 8E. An increase in the TGF-*β* level leads to an increase in the N2-to-N1 ratio, which in turn increase the tumor size. In Fig 8F, we investigate the correlation between N21R and the tumor size. The tumor volume and N21R were calculated from tumor and TAN populations in response to various TGF-*β* stimuli (*G* = 0 − 0.9) with two different initial conditions: *C*(0) = 1, *I*(0) = 2.5 in red asterisk; *C*(0) = 2.5, *I*(0) = 1 in blue circle. The case of the fluctuating *G*(*t*) was marked in black square. As N21R is increased, the tumour volume is increased, leading to a positive correlation between N21R and the tumor size. The neutrophil-to-lymphocyte ratio (NLR) has been highly associated with cancer progression [69, 70], becoming biomarkers for various cancers including non-small-cell lung cancer [71, 72] and cervical cancer [73]. In our simulation, N21R is equivalent to NLR and may be suggested to be a prognostic factor.

See S1 Appendix for analysis of the model. We investigated effects of N1 immunity (*δ*) on cancer cell killing. Depending on various values of *δ*, the killing rate of cancer cells by N1 TANs, time courses of tumor volume in response to fluctuating TGF-*β* may show different patterns: monotonic increase, monotonic decrease, or alternation between growth and shrinkage.

### Dynamic transition to N1-dominant TANs by normalizing the complex immune system

**Fig 9:**
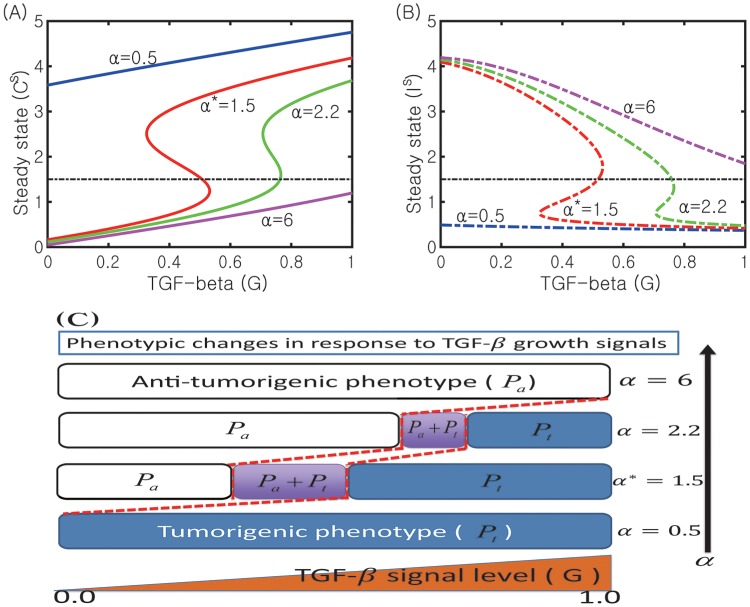
Transition to the N1-dominant environment by increasing *α*. (A, B) Bifurcation curves *G* − *C* (*C*^*s*^ in (A)) and *G* − *I* (*I*^*s*^ in (B)) for various N2 inhibition strengths (*α* = 0.5, 1.5, 2.2, 6.0). (C) The corresponding phenotypic switches between tumorigenic and anti-tumorigenic phases when *α* is varied. Other parameters are fixed as in Table 2. The base case (α* = 1.5) was marked in star (*).

In Fig 9A, we show the sensitivity of *G*-dependent N2 states (*C*^*s*^) to changes in inhibition strength of the N2 module by the N1 module (*α* = 0.5, 1.5, 2.2, 6.0). The corresponding N1 states (*I*^*s*^) are shown in Fig 9B. As *α* is increased, the bifurcation curve (*C*^*s*^, *G*) shifts to the right. This increase in *α* also moves the bi-stability window (*W*_*G*_) to the right and leads to a decrease in the size of the window (|*W*_*G*_|). This indicates that the probability of switching to the anti-tumorigenic phase ($P_{a}$) is increased when *α* is increased. For example, the immune response is already in the $P_{a}$-phase (*C* < *C*^*th*^, *I* > *I*^*th*^) when *G* = 0.6 in the case of the higher *α* value (*α* = 2.2; green curve in Fig 9A) while it is still in the $P_{t}$ phase (*C* > *C*^*th*^, *I* < *I*^*th*^) in the base case (α* = 1.5; red line in Fig 9A). On the other hand, the immune system would initiate the tumor-favoring progress even under low TGF-*β* conditions when *α* is decreased. For example, the system would be in the $P_{t}$-phase for a low TGF-*β* signal (*G* = 0.1) when *α* is lowered (*α* = 0.5; blue curve in Fig 9A) while it should be in the $P_{a}$-phase in the base case (α* = 1.5). We recall that, for the base case (α* = 1.5), the existence of the bi-stability regime *W*_*G*_ illustrates that the TGF-*β* signal can regulate the forward transition from the $P_{a}$-state to the $P_{t}$-state and the recurrent process from the $P_{t}$-state to the $P_{a}$-state. However, when the N2 activity is highly enhanced with the decreased inhibition signal from the N1 complex (a decrease in *α*), the TGF-*β* signal may regulate only forward transition from the $P_{a}$-state to the $P_{t}$-state (one way switch) and a monotonic decrease in TGF-*β* signals does not push the immune system to the anti-tumorigenic phase. The phenotypic changes between $P_{a}$- and $P_{t}$-states for increased or decreased *α* are illustrated in Fig 9C. This mechanism of low *α*’s therefore provides a way of keeping the immune system in a tumorigenic phase. Functional plasticity associated with a decrease in *α* induces a critical immunosuppressive switch to promote aggressive tumor growth and cancer progression [35, 74].

**Fig 10:**
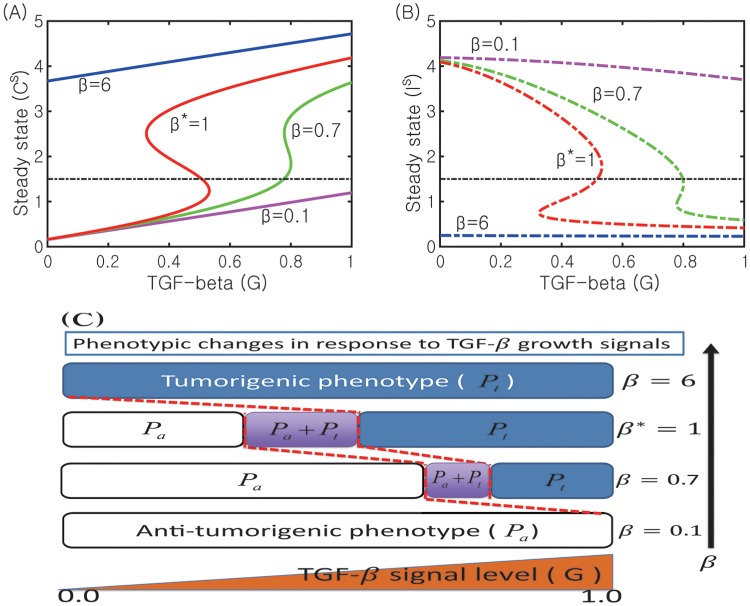
Effect of the inhibition parameter (*β*) on the transitions between N1 and N2 system. (A, B) Bifurcation curves *G* − *C* (*C*^*s*^ in (A)) and *G* − *I* (*I*^*s*^ in (B)) for for various N1 inhibition strengths (*β* = 0.1, 0.7, 1.0*, 6.0). (C) The corresponding phenotypic switches between tumorigenic and anti-tumorigenic phases when *β* is varied. Other parameters are fixed as in Table 2. The base case (β* = 1.0) was marked in star (*).

In Fig 10 we show the effect of the inhibition strength of N1 activities by the N2 module (*β* = 0.1, 0.7, 1.0*, 6.0) on the dynamics of the core control system. As *β* is increased, the bifurcation curves (*G*, *C*) shift to the left (Fig 10A). In contrast to the case of changes in *α* in Fig 9, this increase in *β* reverses its shifting direction *i.e.,* it moves the bi-stability window (*W*_*G*_) to the left and leads to an increase in the size of the window (*W*_*G*_) despite disappearance of the bistable switch for larger *β*. Thus, an increase in *β* induces transient changes from a bistable- to a one-way-switch and to a mono-stability. This indicates that the probability of switching to the tumorigenic phase ($P_{t}$) is increased in response to fluctuating TGF-*β* levels (0 ≤ *G* ≤ 1.0) when *β* is increased. For example, the immune system is already in the tumorigenic phase ($P_{t}$; *C* > *C*^*th*^, *I* < *I*^*th*^) in response to *G* = 0.12 in the case of higher *β*’s (for example, *β* = 6.0; blue curve in Fig 10A) while it should still be in the anti-tumorigenic phase ($P_{a}$; *C* < *C*^*th*^, *I* > *I*^*th*^) in the base case (β* = 1.0; red line in Fig 10A). On the other hand, the immune system would activate the anti-tumorigenic machinery even under high TGF-*β* signal conditions when *β* is decreased. For example, the system would increase probability of switching to the anti-tumorigenic phase by lowering N2 levels for a high TGF-*β* level (*G* = 1.0) when *β* is lowered (*β* = 0.1; cyan curve in Fig 10A) while it should be in the tumorigenic phase in the base case (β* = 1.0). While a decrease in *β* reduces the size of the bi-stability window (|*W*_*G*_|), an increase in *β* reduces the size of the one-way switch window. When N2 activities are highly enhanced with the increased inhibition signal of the N1 module by the N2 complex (an increase in *β*) due to mutual antagonism between N2 and N1 modules, TGF-*β* signals may also regulate only forward transition from $P_{a}$-state to $P_{t}$-state (one way switch) and a monotone decrease in TGF-*β* signals does not push the immune system to the anti-tumorigenic phase. Therefore, this *β*-induced mechanism has a similar effect on keeping high N2 activities as that of a decrease in *α*, providing a way of keeping the immune system in a tumorigenic phase.

Therefore, these results provide a scientific basis for targeting suppressive effect of N2 TANs by normalizing immune activities (increase in *α* or decrease in *β*) within the tumor microenvironment or keeping a favorable balance of the N1/N2 TAN’s properties toward N1 TANs [35]. For example, blood normalization was suggested to be an alternative way of creating anti-tumor TAN microenvironment [75, 76].

### Role of IFN-*β* signaling in regulation of the transition between N1- and N2-dominant system

**Fig 11:**
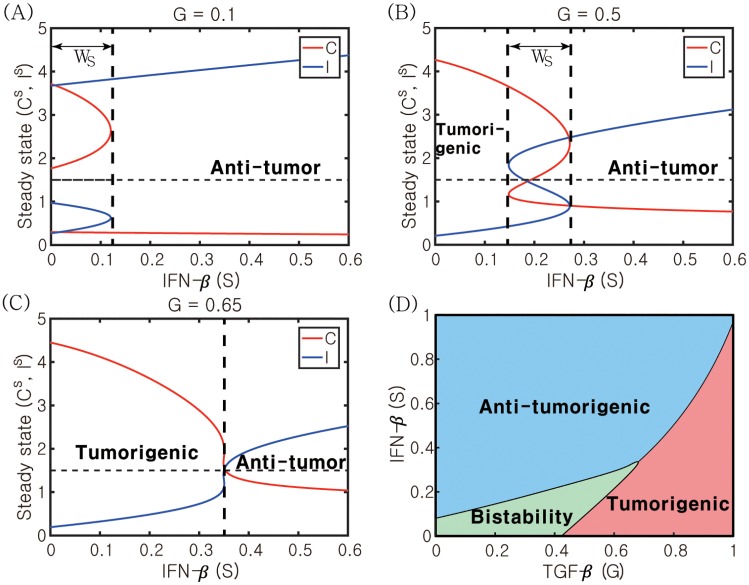
Dynamic change in response to the IFN-*β* signal. (A-C) Activities of N2- and N1-modules in response to IFN-*β* levels (*S*) when the TGF-*β* level is low (*G* = 0.1 in (A)), intermediate (*G* = 0.5 in (B)), and high (*G* = 0.65 in (C)). When the TGF-*β* level is low or intermediate, there exists a bi-stable window (*W*_*S*_ = {*S* ∈ [0, 1]: *S*^*m*^ ≤ *S* ≤ *S*^*M*^}) where both $P_{t}$ and $P_{a}$ coexist. (D) For a constant IFN-*β* signaling, the system transits from $P_{a}$- to $P_{t}$-modes as the TGF-*β* level is increased. On the other hand, for a fixed TGF-*β* level, an increase in *S* induces a transition from $P_{t}$-favoring mode to the $P_{a}$-status. Other parameters are given in Table 2.

In Fig 11, we investigate the role of the IFN-*β* signaling pathway in the phenotypic balance of N2 and N1 complexes for various TGF-*β* levels (*G* = 0.1, 0.5, 0.65). When *G* is low, the system induces a bi-stable system for low IFN-*β* levels and N1-dominant phenotypes in response to intermediate and high IFN-*β* levels (Fig 11A). Therefore, the system is quite sensitive to relatively low IFN-*β* stimuli to induce the anti-tumor microenvironment. When *G* is increased to *G* = 0.5, the bistability window (*W*_*s*_) in Fig 11A moves to the right and the system now can generate tumorigenic, bi-stable, and anti-tumorigenic status in response to low, intermediate, and high IFN-*β* levels, respectively. Therefore, the relative balance between TGF-*β* and IFN-*β* signaling determines the phenotypic preference (Fig 11B). On the other hand, in the presence of high levels of TGF-*β* (*G* = 0.65; Fig 11C), the bistability window disappears and the probability of inducing a N1-dominant microenvironment is decreased, shifting the balance toward the high N2-to-N1 ratio. Fig 11D summarizes the phenotypic representation of the system in response to various TGF-*β* and IFN-*β* stimuli. The majority of the bi-stable region resides in the lower left corner of the *G* − *S* plane. Therefore, the biochemical fluctuation of both TGF-*β* and IFN-*β* levels in the lower regime is likely to generate a selection process of either promoting or suppressing tumor growth based on the initial distribution and phenotypic status of TANs in tumor microenvironment.

### Sensitivity analysis

In the model developed in this paper, there are parameters for which no experimental data are known and they may affect the simulation results. We take all parameters in the model (*r*, *K*, *γ*_1_, *T*_0_, λ, *δ*, *G*, *S*, *k*_1_, *k*_2_, *k*_3_, *k*_4_, *α*, *β*, and *μ*) for sensitivity analysis. We investigated the sensitivity of the activities of N2 and N1 modules at *t* = 10, 100, 200 to these parameters. We have chosen a range for each of these parameters and divided each range into 10,000 intervals of uniform length. For each of the fifteen parameters of interest, a partial rank correlation coefficient (PRCC) value is calculated. PRCC values range between -1 and 1 with the sign determining whether an increase in the parameter value will decrease (-) or increase (+) activities of the N2 and N1 complex, and tumor size at a given time. The sensitivity analysis to be described below was carried out using the method from [77] and Matlab files available from the website of Denise Kirschner’s Lab: http://malthus.micro.med.umich.edu/lab/usadata/.

**Fig 12:**
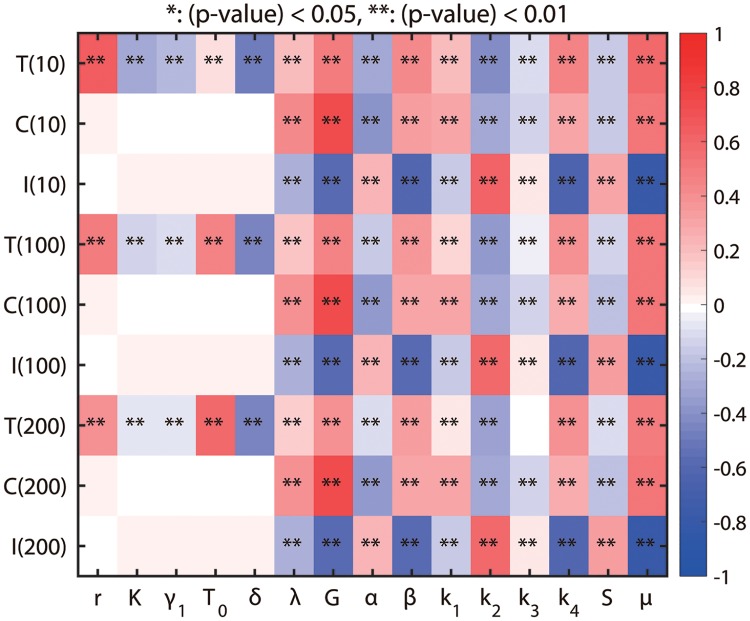
Sensitivity analysis: General Latin Hypercube Sampling (LHS) scheme and partial rank Correlation Coefficient (PRCC) performed on the model. The colors in each sub box indicates PRCC values of the N2 level and N1 activity for model parameters (*r*, *K*, *γ*_1_, *T*_0_, *δ*, λ, *G*, *S*, *k*_1_, *k*_2_, *k*_3_, *k*_4_, *α*, *β*, *μ*) at *t* = 10, 100, 200. The analysis was carried out using the method of [77] with sample size 10000.

Fig 12 shows the sensitivity analysis results for the N2 level and N1 activity, and tumor volume at *t* = 10, 100, 200 for Eqs (5)–(7) *i.e.,* we compute PRCC values for *C*, *I*, *T* at time *t* = 10, 100, 200. We calculate PRCC values and associated *p*-value for fifteen perturbed parameters (*r*, *K*, *γ*_1_, *T*_0_, *δ*, λ, *G*, *S*, *k*_1_, *k*_2_, *k*_3_, *k*_4_, *α*, *β*, *μ*). We conclude that the activity of the N2 complex at *t* = 200 is positively correlated to the parameters λ, *G*, *μ* but is very little correlated (and thus not sensitive) to *r*, *K*, *γ*_1_, *T*_0_, *α*, *β*, *k*_1_, *k*_2_, *k*_3_, *k*_4_, *S*. Thus, in particular, the N2 level will increase significantly if the TGF-*β* signal is increased. Due to mutual antagonism between N1- and N2-dominant system, it is expected to see opposite correlations between N1 activity and those parameters. Indeed, the N1-dominant system is positively correlated to the parameters *k*_2_, *S* but is negatively correlated to the parameters *G*, *β*, *k*_4_, *μ*, *i.e.,* there would be significant reduction in the N1 expression level if the TGF-*β* signaling strength is increased. The tumor volume is positively correlated to the growth rate (*r*) and carrying capacity (*T*_0_) but is negatively correlated to the tumor killing rate by N1 TANs (*δ*).

### Therapeutic approaches: Blocking the N2-dominant system by TGF-*β* inhibitors

**Fig 13:**
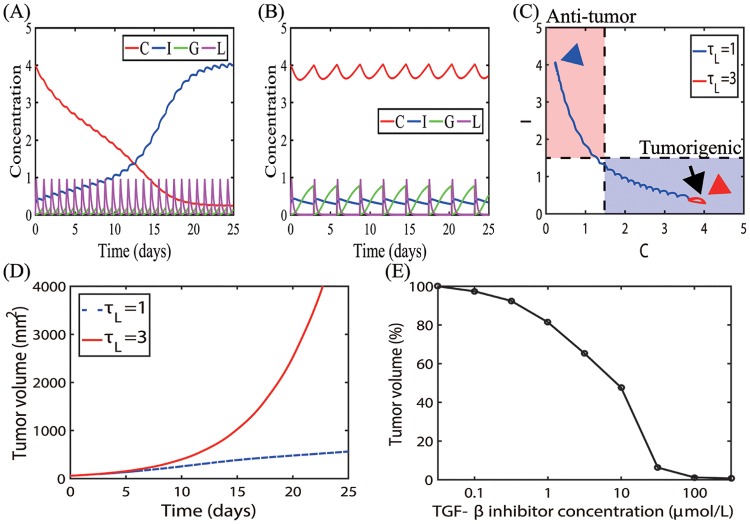
Therapeutic effect of TGF-*β* inhibitor injection. (A, B) Time courses of activity levels of the N2 complex (*C*) and N1 complex (*I*), and concentrations of TGF-*β* (*G*) and TGF-*β* inhibitor (*L*) in response to a periodic injection of the TGF-*β* inhibitor with periods *τ*_*L*_ = 1 (A) and *τ*_*L*_ = 3 (B), respectively. (C) Dynamics of the core N1-N2 system in response to the frequent (*τ*_*L*_ = 1 in (A)) and less frequent (*τ*_*L*_ = 3 in (B)) injections of the TGF-*β* inhibitor in the *C* − *I* plane. (D) Time courses of tumor volume for two different injection protocols in (A, B). (E) Tumor volume (%) at final time (*t* = 25 *days*) in response to various TGF-*β* inhibitor levels (0, 0.02, 0.1, 0.3, 1.0, 3.0, 10, 20, 100 *μmol*/*L*). Initial conditions: *C*(0) = 4.0, *I*(0) = 0.3, *G*(0) = 0, *L*(0) = 0. Initial injection time: *t*_1_ = 0. All other parameters are fixed as in Table 2.

In Fig 13 we investigate the anti-tumor effect of a TGF-*β* inhibitor on tumor growth. We introduce new variables: concentration of TGF-*β* (*G*), instead of a parameter, and TGF-*β* inhibitor concentration (*L*) whose dynamics are described as follows:

$$\frac{dG}{dt}=G_{s}-\mu_{G}G-\gamma_{L}LG,$$ (8)

$$\frac{dL}{dt}=\sum_{i=1}^{N_{L}}L_{s}J_{\left[t_{i},t_{i}+h_{d}\right]}-\mu_{L}L.$$ (9)

Here, *G*_*s*_ is the constant source of TGF-*β*, *μ*_*G*_ is the decay rate of TGF-*β*, *γ*_*L*_ is the degradation rate of TGF-*β* by its inhibitor, *L*_*s*_ is the external periodic source of the TGF-*β* inhibitor on the time intervals [*t*_*i*_, *t*_*i*_ + *h*_*d*_], *i* = 1, …, *N*_*l*_ with the duration *h*_*d*_ and the period *τ*_*L*_ (= *t*_*i*+1_ − *t*_*i*_, *i* = 1, …, *N*_*L*_ − 1; *h*_*d*_ < *τ*_*L*_) between those intervals, *N*_*L*_ is the number of injections, *J*[⋅] is the indicator function (giving 1 and 0 on the given condition), *μ*_*L*_ is the decay rate of the TGF-*β* inhibitor. Eqs (8) and (9) then are coupled with the original Eqs (5)–(7) to investigate the effect of TGF-*β* inhibitors on the transition behaviors between N1 and N2 phenotypes. In Fig 13(A) and 13(B), the dynamic system shows different mode changes as the TGF-*β* inhibitor was injected at the different periods (*τ*_*L*_ = 1, 3). A frequent injection of the inhibitor (*τ*_*L*_ = 1 in Fig 13A) induces a decrease in the TGF-*β* level (*G* < 0.4), resulting in the transition from the initial tumorigenic condition (black arrow in Fig 13C) to the anti-tumorigenic mode (blue arrowhead in Fig 13C). On the other hand, the TGF-*β* level still stays in the upper stable branch (Fig 6) in response to a less frequent injection schedule (*τ*_*L*_ = 3 in Fig 13B), keeping the system in the tumorigenic phase (red arrowhead in Fig 13C). These different cellular compositions in N1- or N2-dominant tumor microenvironment result in fast or slow tumor growth: slow and fast tumor growth in response to frequent (*τ*_*L*_ = 1) and less frequent (*τ*_*L*_ = 3) injection schedules, respectively (Fig 13D). Fig 13E shows the normalized tumor volume at final time (*t* = 25 *days*) for various TGF-*β* inhibitor levels (*L*_0_ = 0 − 100 *μmol*/*L*). As the loading dose is decreased, the anti-tumor efficacy is significantly decreased especially when its concentration is less than 10 *μmol*/*L*. These results are in good agreement with experimental data [38]. Serizawa *et al*. [38] found that gefitinib, a TGF-*β* inhibitor, can effectively kill non-small-cell lung cancer cells, PC-9 (cell lines without erlotinib, an EGFR inhibitor) and PC-9ER1/PC-9ER4 (cell lines with resistance to erlotinib) in *in vitro* experiments [38]. While the overall cell viabilities in these cell lines were significantly decreased in response to the increasing concentrations of gefitinib, the anti-tumor efficacy in low dose of gefitinib (∼0.1*μmol*/*L*) was higher and sensitive in PC-9 cell lines in the response curve. This implies that the higher dose TGF-*β* inhibitor needs to be injected into the tumor in order to maintain the same level of anti-tumor efficacy in the tumor microenvironment.

### Therapeutic strategies by IFN-*β* injection

We investigate the effect of normalizing immune activities on tumor growth by injecting IFN-*β*. We consider the following conventional equations for time-dependent IFN-*β* administration:

$$\frac{dS}{dt}=\sum_{i=1}^{N_{s}}S_{s}J_{\left[t_{i},t_{i}+h_{s}\right]}-\mu_{S}S$$ (10)

where *S*_*s*_ is the injection dose of IFN-*β*, *J*(⋅) is the indicator function. The IFN-*β* is injected over the time interval [*t*_*i*_, *t*_*i*_ + *h*_*s*_], *i* = 1, …, *N*_*s*_ for the time duration *h*_*s*_ and a period *τ*_*s*_ (= *t*_*i*+1_ − *t*_*i*_, *i* = 1, …, *N*_*s*_ − 1), *N*_*s*_ is the number of the IFN-*β* injections.

**Fig 14:**
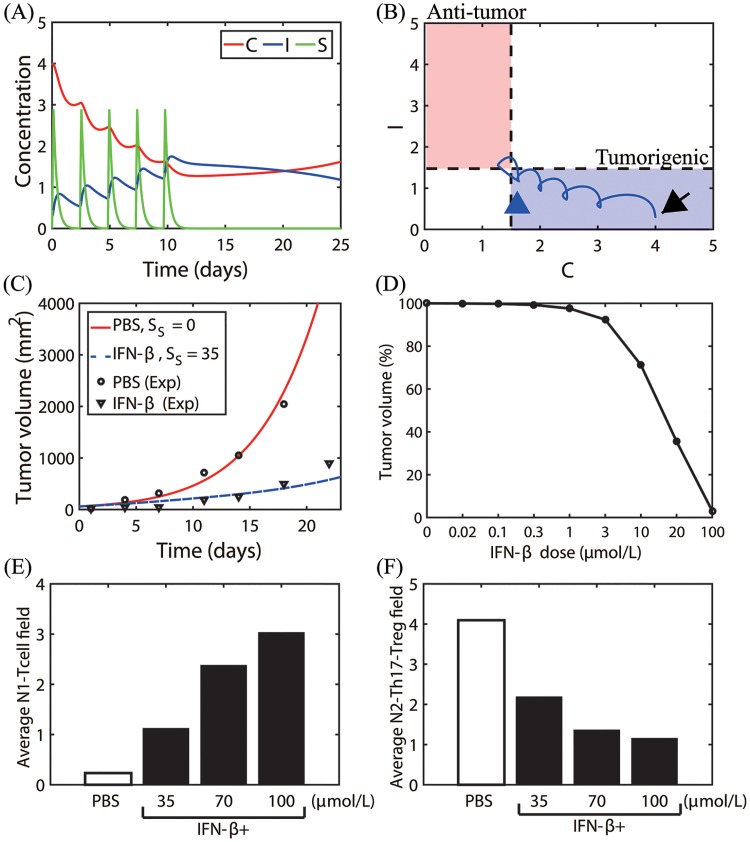
Therapeutic effect of IFN-*β* on tumor growth. (A) Time courses of the N2 complex level (*C*), N1 complex activity (*I*), and concentration of IFN-*β* (*S*) in response to a periodic injection of IFN-*β* on 0, 3, 5, 7, 9 *days*. (B) Dynamics of the core N1-N2 system corresponding to (A) in the *C* − *I* plane. Black arrow = initial position, blue arrowhead = terminal point at *t* = 25 *days*. (C) Time courses of tumor volume in control (PBS) and IFN-*β*^+^ cases: Control (PBS) from the model (red solid line, *S*_*s*_ = 0), IFN-*β* injection case from the model (blue dashed line, *S*_*s*_ = 35), control (PBS) from experiments (empty circle), IFN-*β* injection case from experiments (triangle). (D) Tumor volume (%) at final time (*t* = 25 *days*) in response to various IFN-*β* injection levels (*S*_*s*_ = 0, 0.02, 0.1, 0.3, 1.0, 3.0, 10, 20, 100 *μmol*/*L*). (E, F) Tumor-infiltrating CD8 T cells and Tregs. Average field of N1-Tcell (E) and N2-Th17-Tregs (F) over the time interval [0–12 *days*], in the absence (PBS) and presence of IFN-*β* injections (35,70,100 *μmol*/*L*). Initial conditions: *C*(0) = 4.0, *I*(0) = 0.3, *S*(0) = 0. Initial injection time: *t*_1_ = 0. Parameters used: *G* = 0.4. All other parameters are fixed as in Table 2.

Fig 14A shows the time courses of N2 levels (*C*), N1 activity (*I*), and IFN-*β* level (*S*) in response to intratumoral injections of IFN-*β*. Fig 14B shows the corresponding trajectory of the solution (*C*(*t*), *I*(*t*)) in the *C* − *I* plane. Starting from an initial condition in the tumorigenic phase (black arrow in Fig 14B), the N2-dominant system responds to the injected IFN-*β* in a periodic fashion and slowly transits to the N1-dominant system (pink box in Fig 14B) by decreasing the N2 level and increasing N1 activity (blue arrowhead). Fig 14C shows time courses of tumor volume (*mm*^2^) in the absence (PBS; red solid curve, *S*_*s*_ = 0) and presence (blue dashed curve, *S*_*s*_ = 35) of intratumoral injection of IFN-*β*. IFN-*β* treatment significantly reduces the tumor size through repressing the N2-suppressing immune microenvironment (Fig 14B), which is in good agreement with experimental data. Note that even though the (*C*, *I*) returned back to the tumorigenic phase (blue arrowhead in Fig 14B) after the last IFN-*β* injection on day 9 (Fig 14A), the overall tumor growth is still very slow due to the earlier suppression of the N1-dominant environment, thus normalizing the immune activities against the tumor. The empty circles and triangles in Fig 14C represent experimental results for the LLC1 tumor cell line implanted in C57BL/6 mice in controls (PBS; empty circle) and after IFN-*β* injection (triangles) cases, respectively [36]. In Fig 14D, we show the dose-response curve of IFN-*β* at day 25 after a periodic intratumoral injection of IFN-*β* when *G* = 0.4. IFN-*β* therapy in combination with other anti-tumor drugs [36] has been suggested to be effective in reducing tumor growth [22–25] and stimulating the anti-tumor immune response [37]. Fig 14E shows the average N1-Tcell levels over the time interval [0–12 *days*] in the absence of IFN-*β* (PBS; white bar) and in response to various IFN-*β* doses (35,70,100 *μmol*/*L*; black bars). The corresponding average N2 populations are shown in Fig 14F. In experimental studies [28], the relative population of tumor-infiltrating CD8 T cells were significantly increased in tumor microenvironment in response to IFN-*β* treatment but the Treg population was reduced in non-small cell lung cancer. The model predicts a significant increase in the immune activities (Fig 14E) and dramatic decrease in Treg populations, which is consistent with the experimental results [28].

**Fig 15:**
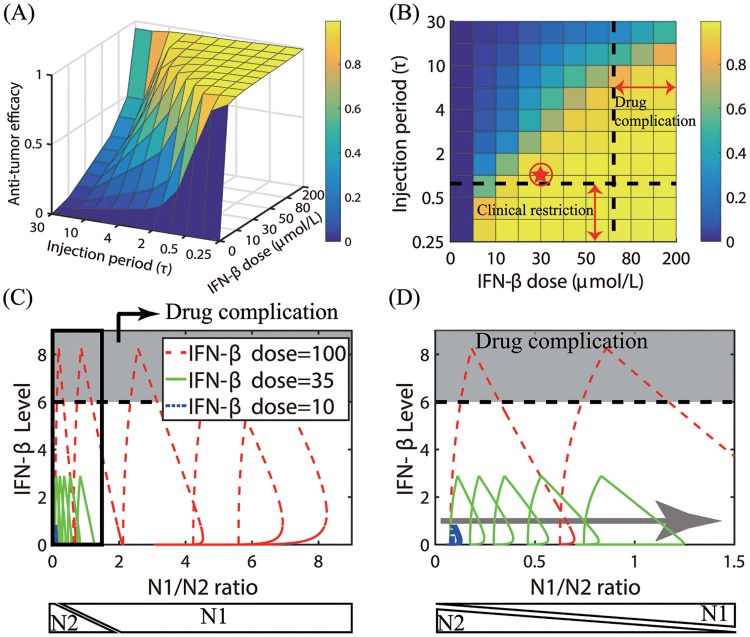
Optimal anti-tumor strategies of IFN-*β* injection. (A) Anti-tumor efficacy for various dose schedules (*τ*_*s*_ = 0.25, 0.33, 0.5, 1, 2, 3, 4, 5, 10, 15, 30 *days*) and injection doses of IFN-*β* (5, 10, 20, 30, 40, 50, 60, 80, 100, 200 *μmol*/*L*). (B) Illustration of anti-tumor efficacy for various schedules in (A) with practical restrictions at a clinic. The optimal dose and schedule are obtained for an intermediate IFN-*β* dose and an injection schedule without drug complication and under clinical restrictions (100 *μmol*/*L*, *τ*_*s*_ = 1 *day*; red asterisk). (C) Trajectories of solutions (Γ(*t*), *S*(*t*)) in the IFN-*β*-Γ plane in response to low (10 *μmol*/*L*, blue dashed line), intermediate (35 *μmol*/*L*, green solid line), and high (100 *μmol*/*L*, red dashed line) doses of IFN-*β* every day (*τ*_*s*_ = 1 *day*). Here, Γ(*t*)(= *I*(*t*)/*C*(*t*)) and *S*(*t*) are the N1/N2 ratio and IFN-*β* concentration, respectively, at time *t*. (D) Enlarged figure from a subpanel (black box on the left) in (C). As the injection strength is increased (10→35→100), the trajectory transits to the N1-dominant system (gray arrow). However, the high dose IFN-*β* may lead to drug complications or extensive associated toxicity (gray zone on the top) [83–86]. N1 and N2 phenotypes on the bottom of panels (C, D) are determined from the N1/N2 ratio.

In Fig 15A, we show anti-tumor efficacy for various dose schedules and injection doses of IFN-*β*. For a fixed IFN-*β* dose, the low (or high) dosing frequency induces N2-dominant (or N1-dominant) tumor microenvironment and thus faster (or slower) tumor growth. For a fixed injection frequency, the system transits from the N2-dominant phase to the N1-dominant phase, thus slower tumor growth, as the injection dose amount (*S*_*s*_) is increased as shown in Fig 14. The mathematical model predicts that the larger injection period (*τ*_*s*_) and smaller dose (*S*_*s*_) would result in the N2-mediated, faster tumor growth while the frequent injection schedule and larger doses would lead to the phenotypic switch to the N1-dominant microenvironment and the greater anti-tumor efficacy. Unfortunately, IFN therapy has been shown to be associated with side effects and significant toxicity in cancer, including neuropsychiatric, constitutional, hepatic, and hematologic effects [78], as well as in multiple sclerosis [79]. Therefore, clinicians would want to optimize the overall administration schedule in order to minimize the dose amount while still avoiding high dose therapy and maintaining sustainable clinical results. In other words, the dose level needs to be minimized in order to avoid drug complication region in Fig 15B. On the other hand, too frequent injections are not desirable due to an increase in administrative costs at a clinic, *i.e.,* avoiding clinical restriction region in Fig 15B. In our simulations, the optimal treatment schedule can be obtained with an intermediate dose of 30 *μmol*/*L* and *τ*_*s*_ = 1 *day* (red circled asterisk in Fig 15B) as explained below. In Fig 15C we show the trajectories of solutions (Γ(*t*), *S*(*t*)) in the IFN-*β*-N1/N2 plane in response to the low (10 *μmol*/*L*, blue dashed line), intermediate (35 *μmol*/*L*, green solid line), and high (100 *μmol*/*L*, red dashed line) doses of IFN-*β* every day (*τ*_*s*_ = 1 *day*). Here, Γ(*t*)(= *I*(*t*)/*C*(*t*)) and *S*(*t*) are the N1/N2 ratio and IFN-*β* concentration, respectively, at time *t*. An enlarged subpanel (black box on the left) in Fig 15C is shown in Fig 15D. As the injection strength is increased (10→35→100), the N1/N2 ratio is increased and the tumor microenvironment transits from N2- to the N1-dominant system (gray arrow), leading to better anti-tumor efficacy (Fig 15A). High doses of IFN-*β* were shown to suppress tumor cell proliferation and promote cell death in both *in vitro* and *in vivo* experiments [80–82]. However, a high IFN-*β* dose also increases IFN-*β* levels, leading to drug complications or extensive associated toxicity (gray zone on the top) [83–86]. Therefore, the injection schedule should be designed to avoid the associated toxicity but still maximize the N1/N2 ratio for optimal clinical outcome.

### Combination therapy

**Fig 16:**
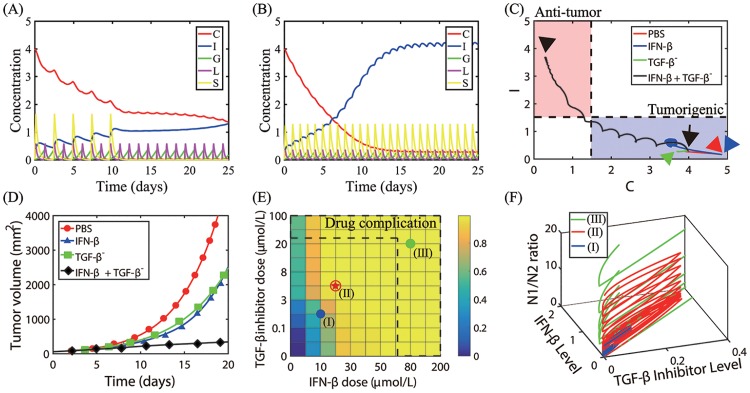
Therapeutic effect of a combination therapy (TGF-*β* inhibitor + IFN-*β*) on tumor growth. (A) Time courses of the N2 (*C*) and N1 complex (*I*) activity, and concentrations of TGF-*β* (*G*), TGF-*β* inhibitor (*L*), and IFN-*β* (*S*) in response to a periodic injection of TGF-*β* inhibitor every day (*τ*_*L*_ = 1 *day*) and IFN-*β* on 0, 3, 5, 7, 9 days. *L*_*s*_ = 8, *S*_*s*_ = 20. (B) Time courses of all variables (*C*, *I*, *G*, *L*, *S*) in response to a periodic injection of both TGF-*β* inhibitor and IFN-*β* every day. *L*_*s*_ = 5, *S*_*s*_ = 15. (C) Trajectories of solutions (*C*(*t*), *I*(*t*)) in response to control (PBS, red) and injections of IFN-*β* only (blue), TGF-*β* inhibitor only (TGF-*β*^−^, green), and combination therapy (IFN-*β* + TGF-*β* inhibitor, black). Black arrow = initial position, arrowheads = terminal points at *t* = 25 *day* for four cases. *L*_*s*_ = 8, *S*_*s*_ = 20. (D) Time courses of tumor volume corresponding to four cases in (C). The combination therapy leads to synergistic effect on killing tumor cells. (E) Anti-tumor efficacy of the combination therapy with various doses of IFN-*β* (0, 5, 10, 20, 30, 40, 50, 60, 80, 100, 200) and TGF-*β* inhibitor (0, 0.02, 0.1, 0.3, 3, 5, 8, 10, 20, 50, 100) at final time (*t* = 25 *days*). The optimal dose schedule (marked in red asterisk) is obtained by avoiding drug complications and minimizing dose levels while maintaining the high anti-tumor efficacy. (F) Trajectories of solutions (*L*(*t*), *S*(*t*), Γ(*t*)) for the low (blue; (I) in (E)), intermediate (red; (II) in (E)), and high (green; (III) in (E)) doses of both TGF-*β* inhibitor and IFN-*β*. Here, Γ(*t*) = *I*(*t*)/*C*(*t*) is the N1/N2 ratio. Initial conditions: *C*(0) = 4.0, *I*(0) = 0.3, *G*(0) = 0, *L*(0) = 0, *S*(0) = 0. Initial injection time: *t*_1_ = 0. All other parameters are fixed as in Table 2.

In this section, we investigate the effect of a combined therapy (TGF-*β*-inhibitor +IFN-*β*) therapy on tumor growth by solving Eqs (5)–(10). Fig 16A shows time courses of the activities of N2 (*C*) and N1 (*I*) modules, and concentrations of TGF-*β* (*G*), TGF-*β* inhibitor (*L*), and IFN-*β* (*S*) in response to a periodic injection of TGF-*β* inhibitor every day (*τ*_*L*_ = 1 *day*) and IFN-*β* on 0, 3, 5, 7, 9 days (*L*_*s*_ = 8, *S*_*s*_ = 20). Injections of both two drugs induce a decrease in *C* and increase in *I*, generating the N1-favoring microenvironment, thus suppressing tumor growth. When these drugs are administered more often (*τ*_*L*_ = 1 *day*, *τ*_*s*_ = 1 *day*), the system quickly switches from the N2-favoring system to the N1-dominant system even with lower doses (*L*_*s*_ = 5, *S*_*s*_ = 15; See Fig 16B). Fig 16C shows the trajectories of solutions (*C*(*t*), *I*(*t*)) in response to control (PBS, red) and injections of IFN-*β* alone (blue), TGF-*β* inhibitor alone (TGF-*β*^−^, green), and combination therapy (IFN-*β* + TGF-*β* inhibitor, black): *L*_*s*_ = 8, *S*_*s*_ = 20. While solutions in control (PBS) and single drug (either IFN-*β* or TGF-*β* inhibitor) stay in the tumorigenic phase (arrowheads (red, blue, green) in the blue box), the solution in the combination therapy (TGF-*β* inhibitor+IFN-*β*) moves to the anti-tumorigenic region (black arrowhead in the pink box). Thus, these dynamics illustrate how the combination therapy may induce the transition from the $P_{t}$-phase to the $P_{a}$-phase even when either TGF-*β* inhibitor or IFN-*β* therapy transits the system to the $P_{t}$-mode. Time courses of the tumor volume corresponding to four cases in Fig 16C are shown in Fig 16D. Thus, the combination therapy leads to synergistic tumor killing effects via switching to the $P_{a}$-phase (Fig 16C). Fig 16E shows anti-tumor efficacy of the combination therapy with various doses of IFN-*β* and TGF-*β* inhibitor at final time (*t* = 25 *days*). For a fixed dose level of either IFN-*β* or TGF-*β* inhibitor, the anti-tumor efficacy is increased in general. However, high doses of either IFN-*β* [78, 85–87] or TGF-*β* inhibitor [88, 89] alone can induce toxicity, resistance and side effects. Therefore, careful dosing strategies need to be developed in order to efficiently deliver IFN-*β* to a tumor and maximize anti-tumor efficacy while minimizing critical, systemic side effects [90, 91]. Therefore, double high doses of those two drugs may not be desirable due to economic costs as well as serious side effects. It has been shown that high doses of TGF-*β* inhibitors may lead to poor response to the combined 5-fluorouracil and IFN*α*-2b therapy in hepatocellular carcinoma patients [92]. The optimal drug dose schedule (red asterisk) can be obtained by avoiding drug complications (dotted region on the upper and right sides) and minimizing dose levels of both drugs while maintaining the overall high anti-tumor efficacy. Fig 16F shows the trajectories of solutions (*L*(*t*), *S*(*t*), Γ(*t*)) for the low (blue; (I) in Fig 16E), intermediate (red; (II) in Fig 16E), and high (green; (III) in Fig 16E) doses of both TGF-*β* inhibitor and IFN-*β*. Here, Γ(*t*) = *I*(*t*)/*C*(*t*) is the N1-to-N2 ratio. While the N1-to-N2 ratios in (II) and (III) are large, the N1-to-N2 ratio is small for low doses of IFN-*β* and TGF-*β* inhibitor (case (I)). Thus, the case (III) in Fig 16E is not desirable because of the high N1-to-N2 ratio.

### Effect of time delays

**Fig 17:**
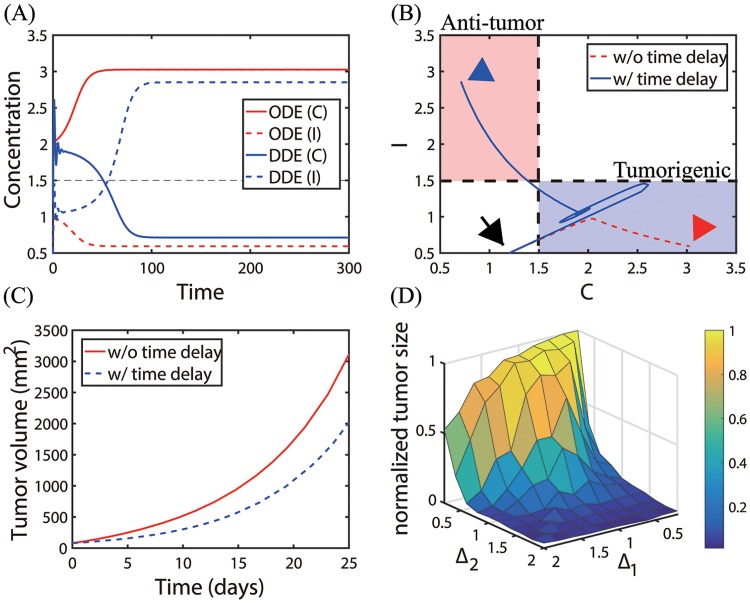
Dynamics of the system with time delays (Δ_1_, Δ_2_) in inhibition of TANs. (A) Time courses of concentrations of two main variables (*C*, *I*) in the absence (ODE, red) and presence (DDE, blue) of time delays (Δ_1_ = 1.0, Δ_2_ = 1.2). (B) Trajectories of solutions (*C*(*t*), *I*(*t*)) in the *C* − *I* plane corresponding to (A). Time delays can push the $P_{t}$-phase to the $P_{a}$-phase. (C) Time courses of the tumor volumes corresponding to (A, B). Time delays can slow down the tumor size by the N1 to N2 transition in (B). (D) Normalized tumor sizes for various strengths of the two time delays (Δ_1_, Δ_2_). Parameters: *S* = 0.2, *G* = 0.4. All other parameters are fixed as in Table 2. Initial condition: *T*(0) = 0.2, *C*(0) = 1.2, *I*(0) = 0.5.

We investigated the effect of time delays on regulation of phenotypic switches and tumor growth. We prescribe time delays in the suppression terms of the N2 and N1 complex. There are many cellular and molecular factors that may affect delayed processes of signaling pathways, apoptosis, and inhibition of neutrophils in the microenvironment [62, 93–96]. This may also be caused by partial blocking of signaling pathways of these TANs by drugs. Recall that the transition probability between $P_{t}$ and $P_{a}$-phases can be modified by the mutual inhibition strength of both N1 and N2 complex (*α*, *β*; Figs 9 and 10). Therefore, we modify Eqs (5) and (6) by introducing time delays in the inhibition terms as follows

$$\frac{dC}{dt}=\lambda_{1}+G+\frac{k_{1}}{k_{3}^{2}+\alpha {\left[I\left(t-\Delta_{1}\right)\right]}^{2}}-C\left(t\right),$$ (11)

$$\frac{dI}{dt}=S+\frac{k_{2}}{k_{4}^{2}+\beta {\left[C\left(t-\Delta_{2}\right)\right]}^{2}}-\mu I\left(t\right),$$ (12)

where Δ_1_, Δ_2_ are the time delays in the inhibitory pathway of the N2 module and suppression of N1 module, respectively (See Fig 4B). Fig 17A shows concentrations of two main variables (*C*, *I*) in the absence (ODE, red) and presence (DDE, blue) of time delays (Δ_1_, Δ_2_) = (1.0, 1.2) with initial condition: *T*(0) = 0.2, *C*(0) = 1.2, *I*(0) = 0.5. The corresponding trajectories of solutions (*C*(*t*), *I*(*t*)) without (red dotted) and with (solid blue) time delays are shown in Fig 17B. Starting with the same initial condition (black arrow), the system adapts to changes in the microenvironment by inducing tumorigenic status (*C* high, *I* low; blue box) and anti-tumorigenic mode (*C* low, *I* high; pink box) in the absence and presence of time delays, respectively. This delay-induced $P_{a}$ mode results in a decrease in the tumor size (Fig 17C). This illustrates that the time delays in suppressing N1 or N2 TANs may stimulate a homeostatic imbalance, resulting in either promotion or suppression of tumor growth. Fig 17D shows the normalized tumor volume at day 25 for various strengths of the two time delays (Δ_1_, Δ_2_). For a fixed Δ_1_, as Δ_2_ is increased, the inhibition strength of the N1 module is decreased and N1 TANs become a dominant phenotype, decreasing the tumor volume. For a fixed Δ_2_, the tumor size is a nonlinear function of Δ_1_, inducing a small tumor for either high or low Δ_1_ but assuming the maximum tumor size for an intermediate Δ_1_. These results illustrate the complex dynamics of the N1/N2 transitions and nonlinear tumor growth in the presence of time delays in mutual inhibitory properties.

**Fig 18:**
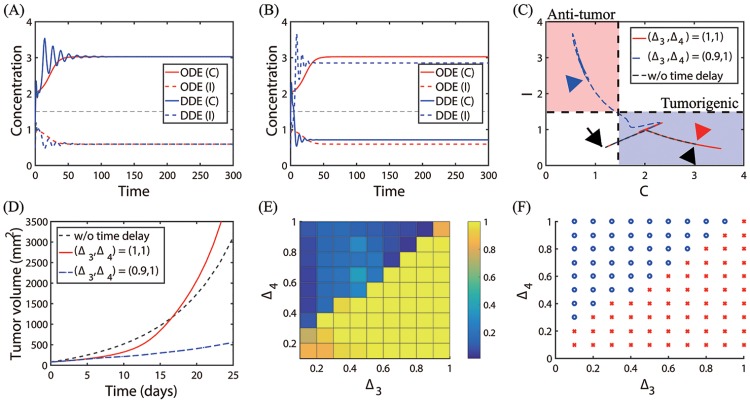
Dynamics of the system with time delays (Δ_3_, Δ_4_) in apoptosis of TANs. (A, B) Time courses of concentrations of two main variables (*C*, *I*) in the absence (ODE, red) and presence (DDE, blue) of time delays: Δ_3_ = 1, Δ_4_ = 1 in (A); Δ_3_ = 0.9, Δ_4_ = 1.0 in (B). (C) Trajectories of solutions (*C*(*t*), *I*(*t*)) without (black dotted) and with ((Δ_3_, Δ_4_) = (1, 1) red solid; (Δ_3_, Δ_4_) = (0.9, 1) blue dashed) time delays in (A-B). (D) Time courses of the tumor volumes corresponding to (C). (E) Normalized tumor sizes for various strengths of the two time delays (Δ_3_ and Δ_4_). (F) Risk map of the time delay-induced transition of TANs from N2 to N1 phenotypes in the Δ_3_ − Δ_4_ plane. Blue circle = switching to the N1-dominant system. Red asterisk = persisting N2-dominant mode. Parameters: *S* = 0.2, *G* = 0.4. All other parameters are fixed as in Table 2. Initial condition: *T*(0) = 0.2, *C*(0) = 1.2, *I*(0) = 0.5.

Now, we investigate the effect of time delays in apoptosis of TANs by modifying Eqs (5) and (6) by introducing time delays in the decay term as follows

$$\frac{dC}{dt}=\lambda_{1}+G+\frac{k_{1}}{k_{3}^{2}+\alpha I^{2}}-C\left(t-\Delta_{3}\right),$$ (13)

$$\frac{dI}{dt}=S+\frac{k_{2}}{k_{4}^{2}+\beta C^{2}}-\mu I\left(t-\Delta_{4}\right),$$ (14)

where Δ_3_, Δ_4_ are the time delays in apoptosis of N2 and N1 modules, respectively. Fig 18(A) and 18(B) show time courses of the N2- and N1-modules in the absence (ODE, red) and presence (DDE, blue) of time delays: (Δ_3_, Δ_4_) = (1, 1) in (A), (Δ_3_, Δ_4_) = (0.9, 1) in (B). Fig 18C shows trajectories of the corresponding solutions (*C*(*t*), *I*(*t*)) in the *C* − *I* plane. The initial position was marked in black arrow. While the presence of time delays (Δ_3_, Δ_4_) = (1, 1) induces initial fluctuations (Fig 18A), the solutions converge to the N2-dominant steady states of ODEs (red arrowhead in Fig 18C). However, a slightly different combination of time delays ((Δ_3_, Δ_4_) = (0.9, 1)) leads to a phenotypic transition from the N2-dominant mode to the N1-dominant phase (blue arrowhead in Fig 18C) despite the initial fluctuation of solutions (blue curves in Fig 18B). Delayed apoptotic cell death leads to different tumor growth dynamics. Fig 18D shows time courses of the tumor volumes corresponding to three cases in Fig 18C. In the presence of time delays (Δ_3_, Δ_4_) = (1, 1), the tumor grows slower than the control (case without time delays) initially but the delay-induced oscillations push the TAN system to the N2-dominant phenotype faster than the control case around *t* = 18 *days*, leading to the larger N2-to-N1 ratio and faster tumor growth (red curve in Fig 18D). On the other hand, in the presence of time delays (Δ_3_, Δ_4_) = (0.9, 1), the tumor grows slower than the control due to strong suppression of the N2 module and enhancement of the N1 module. Fig 18E shows the normalized tumor size at *t* = 25 *days* for various strengths of the two time delays (Δ_3_, Δ_4_ ∈ [0, 1]) with a uniform length of 0.1. A *risk map* of time delay-induced transition of TANs from N2 to N1 phenotypes in the Δ_3_ − Δ_4_ plane is shown in Fig 18F. For a fixed value of Δ_3_, an increase in Δ_4_ results in the higher chance of transition to the $P_{a}$-phase, thus slower tumor growth. On the other hand, for a fixed value of Δ_4_, an increase in Δ_3_ leads to a higher probability of transition to the $P_{t}$-phase and faster tumor growth. To characterize the complex dynamics of the apoptotic cell death mechanism, we introduce the following Δ_4_-to-Δ_3_ ratio:

$$D_{ap}=\frac{\Delta_{4}}{\Delta_{3}}=\frac{\text{time}\;\text{delay}\;\text{in}\;\text{apoptosis}\;\text{of}\;\text{N1}\;\text{TANs}}{\text{time}\;\text{delay}\;\text{in}\;\text{apoptosis}\;\text{of}\;\text{N2}\;\text{TANs}}.$$

When *D*_*ap*_ is large, the delayed apoptotic process of N1 phenotypes and minimal delay in the apoptotic process of N2 phenotypes allow accumulation of more N1 TANs within the microenvironment, pushing the system to the $P_{a}$-phase and effective inhibition of tumor growth. On the other hand, a large delay in the apoptosis of N2 TANs with a relatively small delay in the apoptosis of N1 TANs, (*i.e.,* *D*_*ap*_ ≪ 1), leads to significant accumulation of N2 TANs in the tumor microenvironment, leading to faster tumor growth.

**Fig 19:**
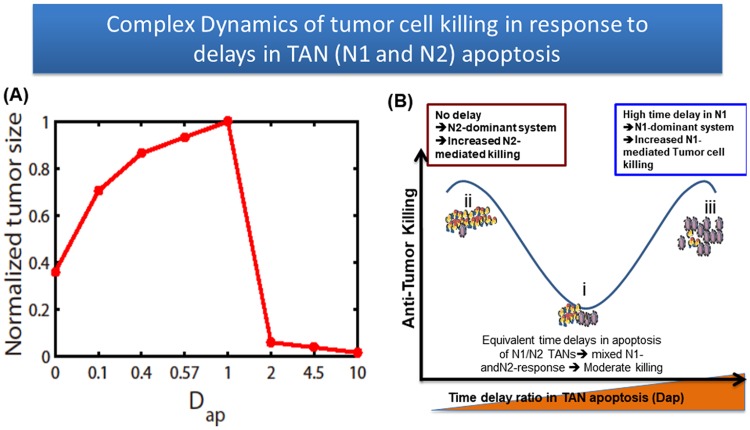
Effect of apoptosis delays of TANs in anti-tumor efficacy. (A) Tumor volume for various time delay ratios of TAN apoptosis (*D*_*ap*_). (B) A model depicting the impact of *D*_*ap*_ on anti-tumor efficacy.

Fig 19A shows the tumor volume for various *D*_*ap*_ at final time *t* = 25 *day*. As *D*_*ap*_ is increased from the control value (*D*_*ap*_ = 1), the tumor size is significantly decreased. On the other hand, a decrease in *D*_*ap*_ also induces slower tumor growth, indicating the maximum tumor growth rate when *D*_*ap*_ = 1. The absence of the time delays in apoptosis signaling also induces slower tumor growth. Fig 19B illustrates a dynamic antitumor killing model with the relative time delay function *D*_*ap*_ as a key parameter. A relatively small delay ratio (*D*_*ap*_ ≪ 1) (basic step i) induces phenotypic perturbations in N1- or N2-responses, which in turn decreases the N2-assisted tumor growth rate. On the other hand, the depletion of time delays in the apoptosis pathways of TANs (step ii) improved antitumor efficacy and suggests that the major antitumor response was likely due to the persistent N2-dominant microenvironment with quick clearance of TANs (Fig 18A). Our study also showed that complementing TANs with adjuvant N1 immunotherapy improved overall antitumor efficacy (step iii), through the facilitation of a robust N1-mediated tumor cell killing response beyond the limits of endogenous N2 TANs. In spite of a typical short half life of neutrophils, delayed apoptosis and accumulation of neutrophils in a tumor environment have been observed in other biological studies [93, 95, 96]. In particular, TANs often have a longer lifespan in the tumor microenvironment due to delayed apoptosis of TANs by modifying its signaling pathways involving FAS, ROS, active Caspase 3, 9, and Bcl-2 family [62]. Our mathematical model predicts that (i) *D*_*ap*_ can be used as a reliable diagnostic measure for aggressiveness of tumor progression; (ii) Anti-tumor efficacy can be improved by increasing the apoptosis decay of N1 TANs or by blocking the accumulation of N2 TANs in tumor microenvironment. In particular, drugs targeting apoptosis pathways of N1 TANs can effectively be used to enhance N1 neutrophil activities to reduce the tumor size.

## Discussion

The immune system protects the host against various types of biological threats. However, it is well established that immune cells show functional plasticity in the context of cancer, and undergo phenotypic changes, promoting tumor growth and metastatic progression [74]. There have been substantial studies to pinpoint fundamental mechanisms through which TANs act on cancer progression [8, 17, 34], and especially via the coordination of chemotaxis-driven recruitment and activation of distinct immune cells to the tumor microenvironment [97–100] as well as immune suppression [74, 101, 102]. A groundbreaking work by Fridlender and colleagues [16] had illustrated that TGF-*β* in tumor microenvironment can generate a functional transition from the proinflammatory neutrophil phenotype (termed “N1”) to the anti-inflammatory phenotype (termed “N2”); [74]. IL-6 also contributes to the formation of the N2 microenvironment [103, 104]. Neutrophil-to-lymphocyte ratio (NLR) was highly associated with progression in many cancer types [69] and has been suggested to be a prognostic factor (or biomarker) for various cancers including colorectal cancer [105, 106], nasopharyngeal carcinoma [107], non-small-cell lung cancer [71], breast cancer [108, 109], hepatocellular carcinoma [110], and melanoma [13]. Recently, it was shown that lung adenocarcinomas can promote bone stromal activity and increase bone mass even without bone metastasis, which in turn enhances tumor growth by remotely supplying tumor-infiltrating neutrophils [111]. However, how the systemic host environment can communicate with a tumor at a remote site is poorly understood.

Since TGF-*β* was identified as a master player that skews differentiation toward the N2 phenotype [16, 18, 19], TGF-*β* inhibitors are suggested to shift the TAN balance toward the N1 phenotype [20, 21]. In cancer research, type I IFNs (*α* and *β*) are substantially studied and tried for cancer therapy due to their anti-tumor capabilities by acting directly on cancer cells and through immunoregulatory characteristic [112]. These Type I IFNs can induce cell cycle arrest and apoptosis in several models [112–114] by acting on several signaling pathways including upregulation of p53 transcription [24]. Type I IFNs are suggested to mediate initial recruitment, active proliferation, phenotypic differentiation, and activation of various immune cells [115–118]. Interestingly, recent evidence from animal models has implicated that IFN-*β* signaling pathways play a critical role in inducing antitumor response in chemotherapy [119] and radiotherapy [68, 120]. Therefore, it is not surprising to see IFN-*β* gene transfer in extensive studies, due to the ability of IFN-*β* to improve (or normalize) immunological response in the tumor microenvironment [121–125]. IFN-mediated neutrophils was shown to upregulate PD-L1 and suppress T-cell proliferation [26].

We have developed a mathematical model by a system of ODEs and DDEs to study the effect of regulatory cytokines (TGF-*β*, IFN-*β*) and delayed apoptotic cell death mechanism on transitions between N1- and N2-dominant system, the N1/N2 mutual antagonism, and their impact on tumor growth (Fig 3). Mathematical analysis showed the complex, nonlinear behaviors of phenotypic switches between N1- and N2-dominant phases in response to fluctuating TGF-*β* and IFN-*β*, and tumor growth patterns (Figs 6–11). In particular, the bi-stability of the N1/N2 system generates a selection process of either promoting or suppressing tumor growth based on the initial distribution and phenotypic status of TANs in a tumor microenvironment (Figs 6 and 11). The microenvironmental pressure of inhibiting the phenotypes also determines the phenotypic switches (Figs 9–10). We also developed therapeutic strategies to control this complex network of TANs for slowing down tumor growth (Figs 13–16). Even though high-dose IFNs were suggested to the only available therapy for relapse-free benefits and overall survival (OS) [126], the administration of high-dose IFN in cancer patients in stage IIb/III needs to be considered carefully [112]. High-dose IFN is not always considered as the first line of treatment option for melanoma patients due to a lack of substantial evidence supporting a long-term OS merit and the extensive associated toxicity [83–86]. Therefore, it is necessary to establish optimal dose and treatment duration of IFNs despite extensive clinical experience [112]. In this study, we investigated the optimal dose schedule of IFN-*β* in the absence or presence of an alternative drug, the TGF-*β* inhibitor (Fig 15). In our mathematical framework, the high N2-to-N1 ratio (equivalent to NLR) was highly associated with faster tumor growth and worse clinical outcomes (Fig 15). Experimental studies [22–25, 36, 38] are in good agreement with the conclusions of the mathematical model. The optimal strategies of reducing the tumor size with minimal side effects [78, 85–89] and maximal antitumor efficacy were explored by using the TGF-*β* inhibitor only, IFN-*β* only, and the combination (TGF-*β* inhibitor + IFN-*β*) therapy (Fig 16).

The life span of neutrophils is strictly regulated in order to maintain tissue homeostasis due to their potential toxicity [127]. These cells are quickly removed from circulation after leaving the bone marrow with a very short half life. However, several proinflammatory cytokines were reported to influence their longevity [128, 129]. There are also many cellular and molecular factors that may affect delays in signaling pathways, apoptosis, and inhibition of neutrophils in the given microenvironment [93–95]. For example, external (smoking and nicotine) and internal (ROS) factors were shown to delay neutrophil apoptosis by suppressing signaling pathways (InsP7, Akt, ROS) in lung diseases such as COPD and lung cancers [93, 95, 96]. Recently, it was also shown that ROS production is reduced in the absence of endogenous IFN-*β*, causing the delayed apoptosis of TANs [62]. Interestingly, a recent study showed that a radiation therapy (RT) can induce the polarization of N1 TANs and reactive oxygen species induced by RT damage tumor tissues, which overall improves anti-tumor efficacy [130]. These microenvironmental factors delay the inhibition and apoptosis of both types of TANs, inducing a complex imbalance between N1 and N2 phenotypes. The mathematical model predicted that various combinations of two time delays in inhibition of signaling pathways within those TANs will induce the relative imbalance between N1 and N2-dominant phases, leading to promotion or suppression of aggressive tumor growth (Fig 17).

We have shown that typical therapy can be exploited by manipulating the delay of apoptosis pathways of both types of TANs. By reducing the relative ratio of apoptosis delay of N1 and N2 TANs, or by increasing time delay of apoptotic pathways of N1 TANs, the efficacy of the antitumor treatment is increased. The mathematical model also predicts that the absence of time delays in apoptosis may allow a greater chance to induce N1 TANs in response to intermediate levels of TGF-*β*, thereby increasing antitumor efficacy (Fig 18). In particular, the model predicted that either strategy, i.e., induction of small (or none) delay of N2 TANs or large delay of N1 TANs, increased the survival rate (Fig 19), even though it is not clear which of the two strategies is clinically better. The answer should also depend on negative side effects associated with the delays of TANs (mixed N1/N2 immune response) and with the injection of apoptosis-targeting drugs (toxicity and inflammation). Our results provide a scientific basis for targeting suppressive effect of N2 TANs by normalizing immune activities (increase in *α* or decrease in *β*) within the tumor microenvironment or keeping a favorable balance of the N1/N2 TAN’s properties toward N1 TANs [35]. While blood normalization was suggested to be an alternative concept of creating an anti-tumor microenvironment including N1 TANs [75, 76], more innovative platforms of normalizing the immune system need to be invented.

In this work, we did not specifically consider many other microenvironmental players such as signaling networks [131], human neutrophil elastase as a therapeutic target in the absence [132, 133] and presence [134] of LPS, endogenous NK immune dynamics [40, 135], angiogenesis from blood vessels [136–138], ECM remodeling [139], or growth factors such as epidermal growth factor (EGF) [140, 141], and CSF-1 [7, 142]. These factors may play critical roles in cancer progression. For example, neutrophil elastin (NE) was shown to indirectly promote the tumor growth by stimulating the PI3K signaling pathways in lung cancer cells [143]. Neutrophil extracellular traps (NETs) were abundant in tumour sections and induce the transformation of B cells [144], contributing to cancer progression [145]. However, another study [146] suggested that the NETs suppressed tumor growth in colonic adenocarcinoma. It is possible that unforeseen microenvironmental factors could have an effect on our approach (governing equations and the many estimated values for mathematical and biological variables). For example, in our model, it is assumed that the tumor microenvironment neutrophils can switch freely from N1 to N2 TANs or from N2 to N1 TANs, given a specific biochemical stimulus. However in vivo there may be unknown biochemical limits to these transitions, such as factors that stabilize one state or another after it has been established. This could be the presence of NETs and associated pro-inflammatory factors. The exact role of this biochemical structural device in regulation of promotion [147–150] or suppression of tumor progression [146] still remains controversial. Our model, however, provides a starting point of the general framework of those key transitions between N1 and N2 TANs in response to known key players. We also plan to investigate the role of the possibly continuous spectrum of the N1 → N2 transition. Further investigation and experimental validation need to be done as more experimental data are available. A multi-scale mathematical model [64, 151–156] could be used to take into account inter and intra-cellular signaling regulation at the microscale level and integrating those to stromal cells and tumor cells at the cellular level. However, the mathematical model in this paper is a first step toward further understanding of lung cancer development in a tumor microenvironment and further experimental investigation. We hope to address these issues in future work.

## Supporting information

S1 Appendix Analysis of the model: Effect of N1 immunity on cancer cell killing. (PDF)
