# Supplementary material for: Role of tumor-associated neutrophils in regulation of tumor growth in lung cancer development: A mathematical model
Source: PLoS One. 2019 Jan 28;14(1):e0211041. doi: 10.1371/journal.pone.0211041 (PMC6349324; doi:10.1371/journal.pone.0211041)
Supplement: S1 Appendix — (PDF) [file pone.0211041.s001.pdf]

Supplementary Material

Analysis of the model

Yangjin Kim, Donggu Lee, Junho Lee, Seongwon Lee, and Sean Lawler

Effect of N1 immunity on cancer cell killing

In Fig. S1, we test the effect of N1 TAN immunity (δ) on tumor growth under a fluctuating TGF-β condition. A time-dependent TGF-β level was assigned for a periodic supply of TGF-β in tumor microenvironment as follows (Fig. S1A):

$$G(t) = \begin{cases} 0.002t + 0.2 & \text{if } 0 < t < 250 \\ -0.002(t-250) + 0.7 & \text{if } 250 \le t < 500 \\ 0.002(t-500) + 0.2 & \text{if } 500 \le t < 750 \\ -0.002(t-750) + 0.7 & \text{if } 750 \le t < 1000. \end{cases}$$

In response to the initial increasing TGF-β level, the trajectory of the solution $C(t)$ follows the lower branches of the hysteresis bifurcation loop (thick gray curve) and jumps to the upper branch (Fig. S1B). However, a decrease in G around $t = 250$ switches the moving direction of the solution and it follows the upper branch in the bi-stability region until it jumps down to the lower branch near the left knee of the hysteresis curve. This flow of the solution $(G(t), C(t))$ was marked in red arrows with initial position on the left lower corner. The blue dashed curve and blue arrows represent the solution $(G(t), I(t))$ and its flow, respectively, in the opposite side. The detailed time courses of solutions $(C(t), I(t))$ in response to the periodic G input are shown in Fig. S1C. The specific N1/N2 phenotypic transitions occur at time $t = t_1, t_2, t_3, t_4$. For example, the initial N1-dominant $\mathbb{P}_a$-status (white region in Fig. S1C) switches to a N2-dominant $\mathbb{P}_t$-mode (pink region in Fig. S1C) in the tumor microenvironment at time $t = t_1$ due to increasing TGF-β levels. The TAN phenotypic ratio, described by $\frac{C}{K+\gamma_1 I}$ in the second term of Eq. (7) in the main text, and cancer cell killing by N1 TANs, described by δIT in the third term of Eq. (7) in the main text, determine either promotion or suppression of tumor growth in the model.

Fig. S1D shows time courses of tumor volumes for various tumor killing rates by N1 TANs in response to fluctuating TGF-β in Fig. S1A: (i) $\delta = 1.0 \times 10^{-4}$ (solid green), (ii) $\delta = 1.0 \times 10^{-3}$ (dashed red), and (iii) $\delta = 1.0 \times 10^{-2}$ (dashed blue). Tumor volumes and slopes of the growth curve of the tumor in those three cases are shown in Fig. S1E and Fig. S1F, respectively. The low killing activities ($\delta = 1.0 \times 10^{-4}$) slow down tumor growth in the N1-dominant regimes (white sectors) relative to enhanced growth phases (N2-dominant, pink sectors). Even though the slopes ($= dT/dt$) of tumor growth in the $\mathbb{P}_t$ phase (green curve in pink regions of Fig. S1F) are much higher than ones in the $\mathbb{P}_a$ phase (green curve in white regions of Fig. S1F), these slopes are still positive in both $\mathbb{P}_t$- and $\mathbb{P}_a$-phases, leading to a monotonic increase in tumor volume (green curve in Fig. S1D). Therefore, N1-mediated immunity is not strong enough to suppress $\mathbb{P}_t$-enhanced tumor growth. When δ is increased ($\delta = 1.0 \times 10^{-4} \to 1.0 \times 10^{-3}$), the tumor cell killing is enhanced by N1 TANs, resulting in shrinkage of the tumor in the $\mathbb{P}_a$-phases (red dashed curve in white regions of Fig. S1D), where N2 TAN activities are suppressed and N1 TAN activities are promoted. Tumor volume is still increasing in the $\mathbb{P}_t$-phase, where N1 TAN activities are suppressed and N2 TAN activities are promoted. This growth/shrinkage pattern is consistent with positive slopes in $\mathbb{P}_t$ regions (red dashed curve in pink regions of Fig. S1F) and negative slopes in $\mathbb{P}_a$ regions (red dashed curve in white regions of Fig. S1F). The alternating growth/shrinkage patterns in response to fluctuating TGF-β ultimately lead to a significant decrease in the overall tumor size at final time ($\delta = 1.0 \times 10^{-3}$ in Fig. S1E). When δ is increased further ($\delta = 1.0 \times 10^{-3} \to 1.0 \times 10^{-2}$), the strong N1 immune responses lead to eradication of the tumor at final time (Fig. S1E). In both $\mathbb{P}_t$- and $\mathbb{P}_a$-phases, the rate of volume changes is negative (blue dashed curve in Fig. S1F), leading to a monotonic decrease in tumor volume at all times (blue dashed curve in Fig. S1D).

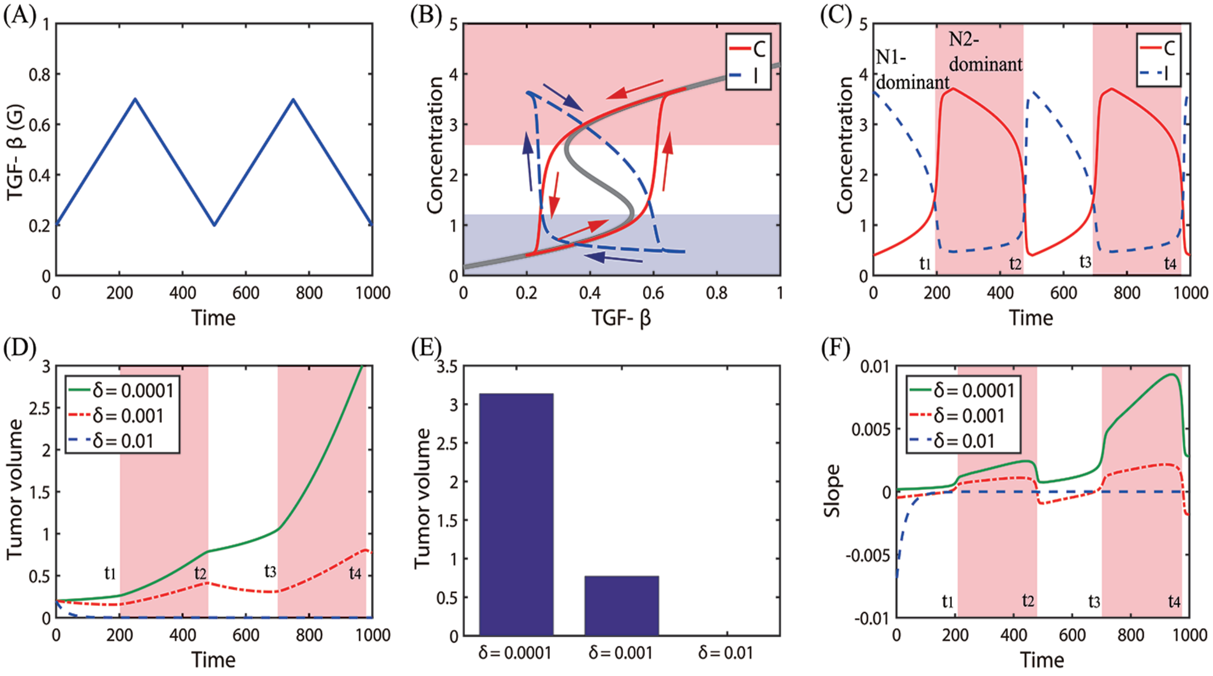

Figure S1. Effect of the N1 immune level (δ) on tumor growth under a fluctuating TGF-β condition. (A) A time-dependent TGF-β level was assigned for a periodic supply of TGF-β in tumor microenvironment. (B) Trajectories of solutions $((G(t), C(t))$ and $(G(t), I(t)))$ in response to TGF-β in (A). The thick gray curve represents the upper and lower branches of steady states ($C - G$ hysteresis bifurcation loop in Fig. 6 in the main text). Red and blue arrows = solution flow direction of C and I, respectively. (C) Time courses of concentrations of C and I in response to periodic G input in (A). The N2-dominant $\mathbb{P}_t$-regions were shaded in pink. (D) Time courses of tumor volumes for various values of δ ($\delta = 1.0 \times 10^{-4}, 1.0 \times 10^{-3}, 1.0 \times 10^{-2}$) in response to the fluctuating TGF-β level in (A). Fluctuating TGF-β levels induce transitions between N1- and N2-dominant phenotypes, leading to transient tumor growth at the transition times (t_1, t_2, t_3, t_4). (E) Tumor sizes at final time for three cases in (D). (F) Time courses of slopes of growth curves for three cases in (D). Initial conditions in (A-F): $C(0) = 0.4$, $I(0) = 3.6$, $G(0) = 0.2$, $T(0) = 0.2$.

This complete eradication of tumor cells with a large δ (e.g., $\delta = 1.0 \times 10^{-2}$) in the system even in the presence of TGF-β-induced tumorigenic microenvironment may happen *in vitro*. However, anti-tumor immune activities are suppressed in tumor microenvironment of many types of cancers including lung cancer. As we illustrated in Fig. 14 and discussion in the main text, tumor growth curves usually show monotonic increase in experiments [1–5]. In this work, parameter δ was chosen to fit the experimental data.

References

1. Catani JPP, Medrano RFV, Hunger A, Valle PD, Adjemian S, Zanatta DB, et al. Intratumoral Immunization by p19Arf and Interferon-beta Gene Transfer in a Heterotopic Mouse Model of Lung Carcinoma. Transl Oncol. 2016;9(6):565–574.

2. Studeny M, Marini FC, Dembinski JL, Zompetta C, Cabreira-Hansen M, Bekele BN, et al. Mesenchymal stem cells: potential precursors for tumor stroma and targeted-delivery vehicles for anticancer agents. J Natl Cancer Inst. 2004;96(21):1593–603.

3. Chiantore MV, Vannucchi S, Accardi R, Tommasino M, Percario ZA, Vaccari G, et al. Interferon-β induces cellular senescence in cutaneous human papilloma virus-transformed human keratinocytes by affecting p53 transactivating activity. PLoS One. 2012;7(5):e36909.

4. Takaoka A, Hayakawa S, Yanai H, Stoiber D, Negishi H, Kikuchi H, et al. Integration of interferon-alpha/beta signalling to p53 responses in tumour suppression and antiviral defence. Nature. 2003;424(6948):516–23.

5. Zhang F, Sriram S. Identification and characterization of the interferon-beta-mediated p53 signal pathway in human peripheral blood mononuclear cells. Immunology. 2009;128(1 Suppl):e905–18.
